# The *Xanthomonas citri* Reverse Fitness Deficiency by Activating a Novel β-Glucosidase Under Low Osmostress

**DOI:** 10.3389/fmicb.2022.887967

**Published:** 2022-05-02

**Authors:** Kaihuai Li, Jinxing Liao, Ming Wei, Shanxu Qiu, Weiyin Wu, Yancun Zhao, Haihong Wang, Qiongguang Liu, Fengquan Liu, Changqing Chang

**Affiliations:** ^1^College of Plant Protection, Integrate Microbiology Research Center, South China Agricultural University, Guangzhou, China; ^2^Guangdong Laboratory for Lingnan Modern Agriculture, Guangzhou, China; ^3^Institute of Plant Protection, Jiangsu Academy of Agricultural Sciences, Nanjing, China; ^4^College of Life Sciences, South China Agricultural University, Guangzhou, China

**Keywords:** low osmostress, physiological deficiencies, β-glucosidase, *Xanthomonas citri* pv. *citri*, bacterial cell growth

## Abstract

Bacteria can withstand various types of environmental osmostress. A sudden rise in osmostress affects bacterial cell growth that is countered by activating special genes. The change of osmostress is generally a slow process under the natural environment. However, the collective response of bacteria to low osmostress remains unknown. This study revealed that the deletion of *phoP* (Δ*phoP*) from *X. citri* significantly compromised the growth and virulence as compared to the wild-type strain. Interestingly, low osmostress reversed physiological deficiencies of *X. citri phoP* mutant related to bacterial growth and virulence. The results also provided biochemical and genetic evidence that the physiological deficiency of *phoP* mutant can be reversed by low osmostress induced β-glucosidase (BglS) expression. Based on the data, this study proposes a novel regulatory mechanism of a novel β-glucosidase activation in *X. citri* through low osmostress to reverse the fitness deficiency.

## Introduction

Bacteria can grow under various environmental osmostress conditions (Record et al., 1998). The exopolysaccharide component of the matrix such as biofilms increases osmotic pressure, which inhibits the biofilm synthesis related gene expression (Rubinstein et al., 2012; Yan et al., 2017). Sudden hyperosmotic stress causes dynamic changes in the cell growth, subcellular distribution of coiled-coil cytoskeletal proteins for cell wall assembly, nucleoid condensation, and turgor pressure (Fuchino et al., 2017). However, the collective response of bacteria to low osmostress remains unknown.

*Xanthomonas citri* pv. *citri* is a Gram-negative bacterium, which causes the canker disease in citrus species, has become a global issue in citrus growing areas by damaging the quality and yield of the citrus (Brunings and Gabriel, 2003; Graham et al., 2004). At present, resistant varieties and effective management methods against this disease are not available.

Two-component system PhoP/PhoQ is one of the main factors regulating the virulence of Gram-negative bacteria (Prost and Miller, 2008). PhoQ is an inner membrane-bound receptor histidine kinase (HK). After detecting environmental signals of Mg^2+^, Ca^2+^, low pH or cationic antimicrobial peptides (AMPs), the PhoQ activates its autokinase activity by phosphorylating a conserved histidine residue (His) and then transfers the phosphoryl group to PhoP, a cytosolic response regulator (RR), through transcription factor activity (Choi and Groisman, 2017; Qadi et al., 2017). The activated PhoP modulates the transcription of downstream genes by directly binding to their *cis*-regulatory elements (CREs) (Supplementary Figure 1A; Garcia Vescovi et al., 1996; Groisman and Mouslim, 2006). Global regulator PhoP is necessary for the motility, biofilm formation, exoenzyme production, and virulence of *X. citri* (Wei et al., 2019).

This study demonstrates the regulation of PhoP in the two-component system PhoP/PhoQ, which contributes to *X*. *citri* growth and virulence. Low osmostress was also found to reverse the physiological deficiencies of *X. citri phoP* mutant related to growth and virulence. Furthermore, this study also proposed a novel regulatory pathway to show that low osmostress reverses the fitness disadvantage of *X*. *citri phoP* mutant strain by activating a novel β-glucosidase (BglS).

## Results

### XAC4022–XAC4023 of *Xanthomonas citri* Encode PhoQ-PhoP Orthologs

Phylogenetic analysis and similarity search revealed that XAC4022-XAC4023 of *X. citri* pv. *citri* strain 306 are the PhoQ-PhoP orthologs of Gram-negative bacteria (Supplementary Figures 1B,C). XAC4022–XAC4023 proteins of *X. citri* share the highest similarity with the PhoP-PhoQ sequences of *Escherichia coli*, *X. campestris* pv. *campestris*, or *pseudomonas aeruginosa*. PhoQ usually contains an N-terminal periplasmic sensor region surrounded by two transmembrane helices. PhoP is an OmpR-family transcription factor (TF) with an N-terminal receiver domain as the phosphoryl acceptor (Response_reg) and a C-terminal helix-turn-helix region (Trans_reg_C) that binds double-stranded DNA (Supplementary Figure 1A). Protein sequence alignments indicated that XAC PhoQ and PhoP shared 77 and 57% identical residues with *P. aeruginosa* PhoP, respectively (Qadi et al., 2017). *X. citri* PhoQ and PhoP shared 95% and 100% identity with their close relative *X. campestris*, respectively (Peng et al., 2017; Supplementary Figure 1C).

### Deletion of *phoP* Affect *Xanthomonas citri* Growth and Low Osmostress Recover Growth Deficiency *of phoP* Mutant

**FIGURE 1:**
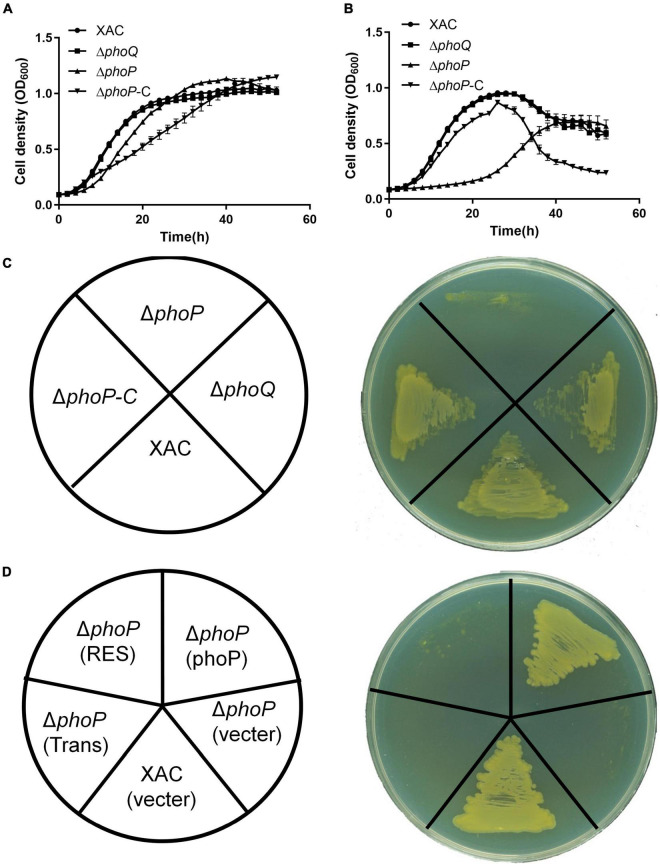
Inactivation of *phoP* reduces the growth of *X. citri* strains. **(A)** Growth curves of bacterial strains in rich YEB medium. **(B)** Growth curves of bacterial strains in rich NYG medium. **(C,D)** Growth of bacterial strains on rich NYG medium plates. OD_600_ of cultures was monitored using a Bioscreen-C Automated Growth Curves Analysis System (Oy Growth Curves FP-1100-C, Helsinki, Finland). Trans and Res, respectively, represent the Trans_reg_C and response_reg domains of PhoP. Error bars mean ± standard deviation (*n* = 3). All experiments were repeated three times with similar results.

Two knockout strains Δ*phoQ* and Δ*phoP* with deleted *phoQ* (XAC4022) and *phoP* (XAC4023) genes were constructed by a two-step homologous recombination approach to identify the physiological functions of PhoQ and PhoP in *X. citri*. The growth of Δ*phoQ* and Δ*phoP* mutant strains was tested in rich medium YEB and NYG. The growth of the *phoP* mutant strain was significantly slower than the wild-type strain in rich medium NYG (Figures 1A–C). *X. citri* growth in rich medium YEB and NYG remained unaffected with the deletion of *phoQ* (Figures 1A–C). To further determine the importance of two key domains (Response_reg and Trans_reg_C) of *phoP*, complemented vectors carrying two domains were constructed that complemented *phoP* mutant strain (named *phoP*-C). The results showed that two single domains could not complement the *phoP* mutant growth defect. The *phoP* mutant strain was only complemented in the presence of both domains (Response_reg and Trans_reg_C) (Figure 1D). These results suggest that PhoP is critical for the growth of *X. citri* and requires the presence of two domains for its biological function.

**FIGURE 2:**
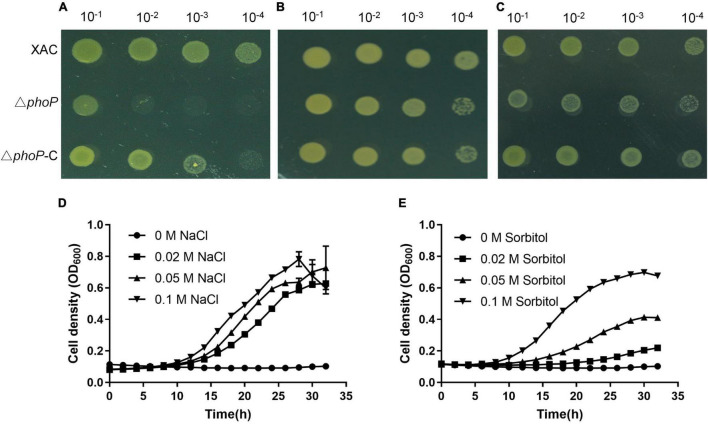
Enhanced growth of *phoP* mutant under low osmostress. **(A)** Growth of bacterial strains on rich NYG medium plates. **(B)** Growth of bacterial strains on rich NYG medium plates supplemented with 0.05 M NaCl. **(C)** Growth of bacterial strains on rich NYG medium plates supplemented with 0.05 M sorbitol. **(D)** Growth curves of *phoP* mutant strain in rich NYG medium supplemented with 0 M NaCl, 0.02 M NaCl, 0.05 M NaCl, or 0.1 M NaCl, respectively. **(E)** Growth curves of *phoP* mutant strain in rich NYG medium supplemented with 0 M sorbitol, 0.02 M sorbitol, 0.05 M sorbitol, or 0.1 M sorbitol, respectively. OD_600_ of cultures was monitored using a Bioscreen-C Automated Growth Curves Analysis System (Oy Growth Curves FP-1100-C, Helsinki, Finland). Error bars, mean ± standard deviation (*n* = 3). All experiments were repeated three times with similar results.

*X. citri* was subjected to various environmental stresses to investigate the effects of PhoP. The growth of *phoP* mutant strain was also tested on NYG plates under various environmental stresses. Interestingly, low osmostress (0.05 M NaCl or Sorbitol) was noted to promote the growth of *phoP* mutant on NYG plates (Figures 2A–C). To further confirm this phenomenon, the effects of different concentrations of NaCl and sorbitol on the growth of *phoP* mutant were studied under liquid conditions. The results depicted that the addition of NaCl and sorbitol promoted the growth of *phoP* mutant under liquid culture conditions (Figures 2D,E). We further confirmed that the addition of KCl or NH_4_Cl also promoted the growth of the *phoP* mutant strain (Supplementary Figure 2). Low osmostress also promoted the growth of *phoP* mutant strain under acidic (pH 5.0) and alkaline (pH 8.0) conditions (Supplementary Figure 3). Therefore, sodium chloride (NaCl) was used as a single factor to study the mechanism of low osmostress on the physiological characteristics of the *phoP* mutant strain.

### Sodium Chloride Increases *phoP* Mutant Strain Virulence

**FIGURE 3:**
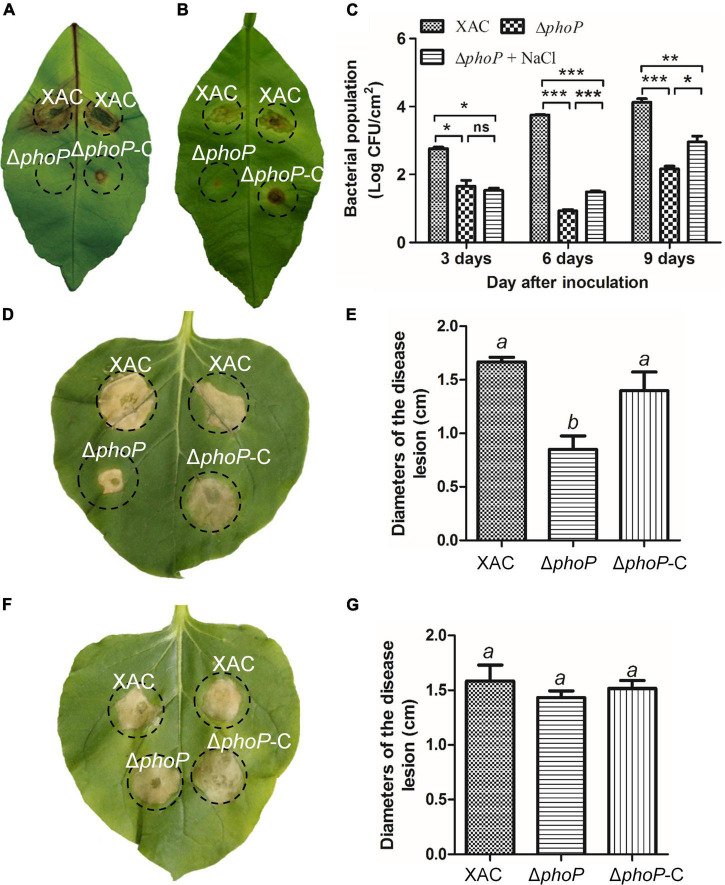
Increase in *phoP* mutant virulence under low osmostress. **(A)** *Citrus* leaves infiltrated with bacterial suspension [5 × 10^8^ colony-forming units (CFU)/mL]. **(B)** *Citrus* leaves infiltrated with bacterial suspension [5 × 10^8^ colony-forming units (CFU)/mL] supplemented with 0.05 M NaCl. **(C)** Bacterial strains were injected into plant leaves using a 1 ml syringe. Bacterial colonies were isolated from leaf disks and counted by serial dilution after inoculation. Each value represents the average number of three-leaf disks. **(D,E)** Effects on the hypersensitive response (HR) of *Nicotiana benthamiana* induced by bacterial suspension. **(F,G)** Effects on the hypersensitive response (HR) of *Nicotiana benthamiana* induced by bacterial suspension supplemented with 0.05 M NaCl. Error bars, means ± standard deviations (*n* = 3). *P < 0.05, **P < 0.01, ***P < 0.001, assessed by one-way ANOVA. All experiments were repeated three times with similar results.

Previous studies have reported the attenuated virulence of *phoP* defective mutant in *E. coli* and *Salmonella* (Coornaert et al., 2013; Ren et al., 2016; Choi and Groisman, 2017). To investigate the role of PhoP in virulence, *X. citri* wild-type and *phoP* mutant strain in *Nicotiana benthamiana* and *Citrus* leaves infection models were also tested. As expected, the deletion of *phoP* reduces bacterial virulence. The infection of the host plant sweet orange (*Citrus sinensis*) with wild-type strain and complemented strain (*phoP*-C) produced canker disease symptoms whereas *phoP* mutant strain caused less hypertrophy and hyperplasia, and failed to produce water-soaking and necrosis symptoms in the susceptible *Citrus* host (Figure 3A). During previous studies, we noted that sodium chloride promoted the growth of the *phoP* mutant strain (Figure 1). Similarly, to test this possibility of enhanced *phoP* mutant strain virulence by sodium chloride, *phoP* mutant strain virulence was evaluated in a sodium chloride environment. *Citrus* leaves were infiltrated with a bacterial suspension supplemented with 0.05 M NaCl, which significantly increased the virulence of the *phoP* mutant strain (Figure 3B). The bacterial population in the plant was also measured. After inoculation, the bacterial population of the *phoP* mutant remarkably decreased as compared to the wild-type strain. However, a significant increase in the population of *phoP* mutant was found when supplemented with 0.05 M NaCl (Figure 3C).

PhoP contribution to hypersensitive response (HR) induction in the non-host tobacco plant (*N. benthamiana*) was also observed. The wild-type strain produced an average lesion diameter of 1.67 cm on *N. benthamiana* after 5 days of inoculation (Figures 3D,E). Deletion of *phoP* significantly reduced the average lesion diameter (0.85 cm) (Figures 3D,E), but complementation with wild-type *phoP* fully restored the HR of *phoP* mutant strain against non-host tobacco plant having an average lesion diameter of 1.4 cm (Figures 3D,E). The results indicated that *phoP* contributes to HR induction in the non-host tobacco plant (*N benthamiana*). Similarly, sodium chloride also promoted the HR of *phoP* mutant strain against non-host tobacco plants (Figures 3F,G). These results suggest that low osmostress is critical for increasing the pathogenicity and HR of the *phoP* mutant strain.

### Low Osmostress Facilitates *phoP* Mutant Strain Fitness Deficiency

The *phoP* mutant showed severely impaired growth and the low osmostress conditions recovered its growth. To investigate the role of low osmostress in *phoP* mutant physiology, several pathogenicity-related virulence factors, swimming motility and against the H_2_O_2_ of *phoP* mutant strain and *phoP* mutant strain supplementing 0.05 M NaCl were evaluated during this study.

Initially, the activity of extracellular enzymes (cellulase, amylase, and protease) was tested. *phoP* mutant had significantly reduced production of extracellular cellulase and amylase, but exhibited slightly higher protease activity than *X. citri* wild type strain on NYG medium (Supplementary Figure 4). The role of low osmostress in *phoP* mutant swimming motility was observed. The results revealed that the deletion of *phoP* abolished swimming motility in *X. citri* (Supplementary Figures 5A,B), and that was reversed by supplementing 0.05 M NaCl (Supplementary Figure 5C).

Bacterial pathogens must overcome the antimicrobial oxidative burst of the host to survive, replicate, and disseminate throughout the host (Wan et al., 2017). To investigate the role of PhoP against the oxidative burst of the host, the growth of *phoP* mutant strain was evaluated on NYG plates under 0.1 mM H_2_O_2_ concentrations stress. The results showed similar growth rates of *X. citri* wild-type strain and complemented strain (*phoP*-C) under the stress conditions. Nevertheless, the *phoP* mutant strain was more sensitive to 0.1 mM H_2_O_2_ concentrations than the wild-type strain (Supplementary Figures 5D,E). The data suggested that PhoP facilitated *X. citri* to counter oxidative stress. The role of osmostress in promoting the survivability of *phoP* mutant strain under oxidative stress was also studied. 0.05 M NaCl significantly enhanced the survivability of *phoP* mutant against antimicrobial oxidative burst (Supplementary Figures 5F,G). These results suggest that the deletion of *phoP* significantly compromised fitness, include exoenzymes, virulence, swimming motility, and response to oxidative stress, and the low osmostress rescued these deficiency.

### Co-regulation of Genes by PhoP and Low Osmostress

During the above-mentioned studies, we found that low osmostress can restore multiple physiological defects caused by *phoP* mutant, including cell growth, virulence, swimming motility and against oxidative stress. Transcriptome analyses (RNA-Seq) of wild-type and *phoP* mutant strain were performed to investigate the role of low osmostress and PhoP in the *X. citri* gene regulation. The results revealed that in comparison to the wild-type strain, 348 genes of *phoP* mutant strain were differentially altered at the transcription level. 192 genes were noted to be up-regulated whereas 156 genes were down-regulated (Supplementary Figure 6A and Supplementary Table 3). The transcriptomes of the *phoP* mutant strain and *phoP* mutant supplemented with 0.05 M NaCl or 0.05 M sorbitol were also analyzed using RNA-Seq. Differential gene expression analysis showed an increase of 266 and a decrease of 122 genes in *phoP* mutant supplemented with 0.05 M NaCl as compared to their expression in the *phoP* mutant strain. In the case of *phoP* mutant supplemented with 0.05 M sorbitol, an increase in 9 genes and decrease in 3 genes was observed as compared to their expression in *phoP* mutant strain (Supplementary Figures 6B,C and Supplementary Tables 4, 5). Further bioinformatics analyses showed that the products of these differentially expressed genes belonged to three major functional categories involved in the cellular component, molecular function, and biological process. In addition, each major category further contains diverse sub-functional groups (Supplementary Figures 6A–C). Transcriptome profiles were also compared using Venn diagrams, which showed an overlap of 2 differentially expressed genes in *phoP* mutant as compared to the wild-type strain and *phoP* mutant supplemented with sorbitol and NaCl. These two genes were annotated as XAC1448: β-glucosidase (BglS) and XAC2312: membrane protein (Supplementary Figure 6D).

### The Role of β-Glucosidase in Recovering *phoP* Mutant Fitness Under Low Osmostress

**FIGURE 4:**
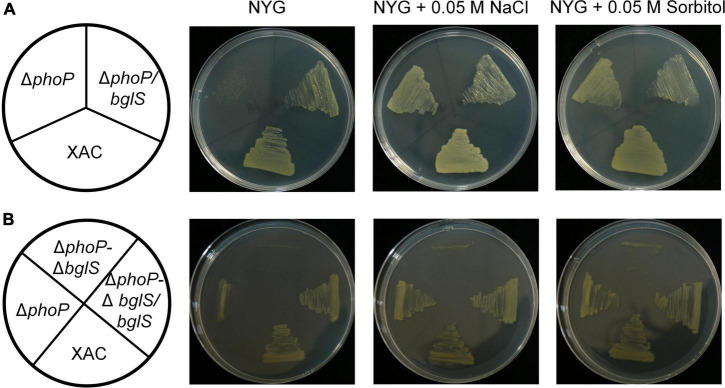
Critical role of BglS in reversing the growth disadvantage of *phoP* mutant under low osmostress. **(A,B)** Growth of bacterial strains on rich NYG medium plates or NYG medium plates supplemented with 0.05 M NaCl or 0.05 M sorbitol.

To investigate the role of *bglS* and XAC2312 in reversing the fitness deficiency through low osmostress, *bglS* and XAC2312 were overexpressed in *phoP* mutant. The results depicted that plasmid-based overexpression of *bglS* significantly promoted the growth of *phoP* mutant strain in NYG plates (Figure 4A) whereas overexpression of XAC 2312 could not significantly reverse the poor growth of *phoP* mutant (Supplementary Figure 7A).

To identify the physiological functions of BglS and XAC2312 response in *X. citri* to low osmostress, the double-mutant strains Δ*phoP-*Δ*bglS* and Δ*phoP-*Δ2312 were constructed by a two-step homologous recombination approach, and their growth was tested on plain NYG and modified NYG plates supplemented with 0.05 M NaCl or 0.05 M sorbitol. The deletion of *bglS* significantly impaired the growth of *phoP* mutant as compared to the wild-type strain, and the low osmostress cannot individually promote the growth of Δ*phoP-*Δ*bglS* mutant on NYG plates (Figure 4B). The growth of the Δ*phoP-*Δ2312 mutant remained similar to the *phoP* mutant under a low osmostress environment (Supplementary Figure 7B). The growth defect phenotypes of Δ*phoP-*Δ*bglS* mutants were restored to wild-type levels by introducing *bglS* into Δ*phoP-*Δ*bglS* (Figure 4B). However, overexpression of XAC2312 in the Δ*phoP-*Δ2312 mutants could not produce a substantial difference between the mutant and complemented strains of *X. citri* in terms of growth (Supplementary Figure 7B). These results suggest that only BglS can reverse the poor growth of the *phoP* mutant.

### The Role of β-Glucosidase in Recovering Swimming Motility of *phoP* Mutant Under Low Osmostress

**FIGURE 5:**
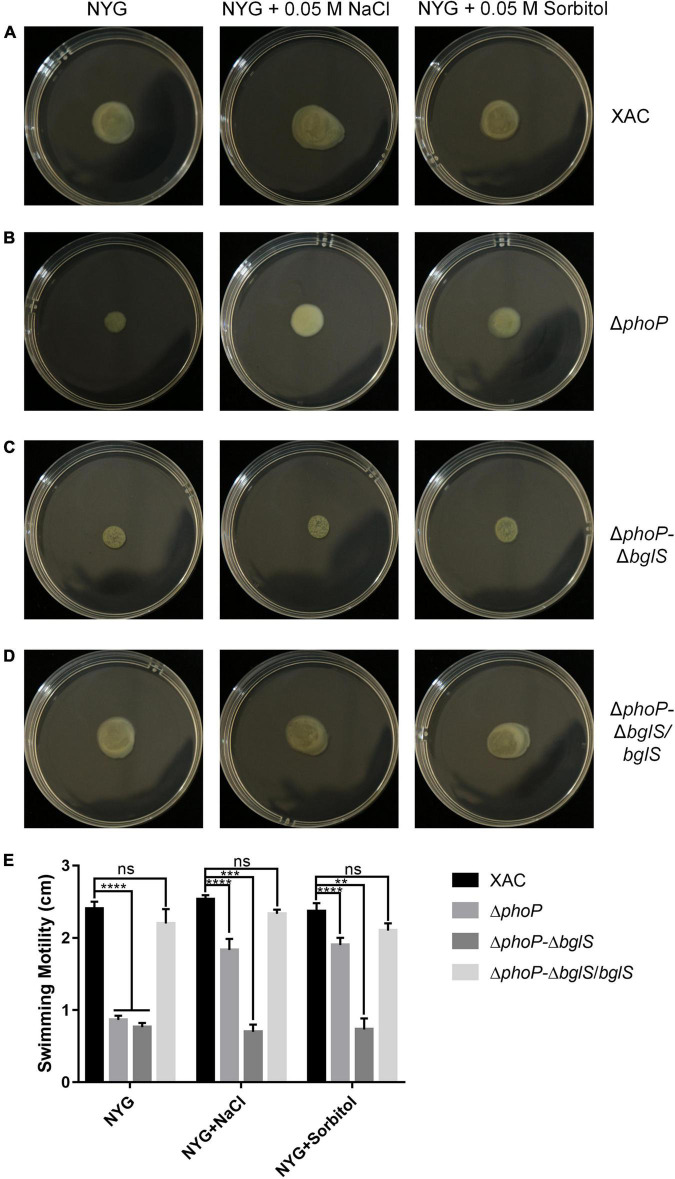
Critical role of BglS in reversing the swimming motility disadvantage of *phoP* mutant. **(A)** Swimming motility of *X. citri* wild-type strain on rich NYG medium plates or NYG medium plates supplemented with 0.05 M NaCl or 0.05 M sorbitol. **(B)** Swimming motility of *phoP* mutant strain on rich NYG medium plates or NYG medium plates supplemented with 0.05 M NaCl or 0.05 M sorbitol. **(C)** Swimming motility of *phoP* and *bglS* double mutant strain (Δ*phoP*-Δ*bglS*) on rich NYG medium plates or NYG medium plates supplemented with 0.05 M NaCl or 0.05 M sorbitol. **(D)** Swimming motility of *bglS* overexpression in *phoP* and *bglS* double mutant strain (Δ*phoP*-Δ*bglS/bglS*) on rich NYG medium plates or NYG medium plates supplemented with 0.05 M NaCl or 0.05 M sorbitol. **(E)** Statistical analysis BglS role in reversing the swimming motility disadvantage of *phoP* mutant. Error bars, means standard deviations (*n* = 3). **P < 0.01, ***P < 0.001, and ****P < 0.0001, assessed by one-way ANOVA. All experiments were repeated three times with similar results.

The above findings confirmed that BglS can reverse the growth deficiency of the *phoP* mutant. To investigate the role of BglS in the swimming motility of *X. citri*, swimming motility growth of Δ*phoP-*Δ*bglS* was evaluated on plain NYG and modified NYG plates supplemented with 0.05 M NaCl or 0.05 M sorbitol. The deletion of *bglS* significantly impaired the swimming motility in *phoP* mutant and low osmostress could not recover Δ*phoP-*Δ*bglS* mutant swimming motility on NYG plates (Figures 5A–C,E). Contrarily, the overexpression of *bglS* in the Δ*phoP-*Δ*bglS* mutants reversed the swimming motility deficiency of *phoP-*Δ*bglS* mutant (Figures 5D,E).

### Global Transcriptional Regulator PhoP Positively Regulated β-Glucosidase Expression

**FIGURE 6:**
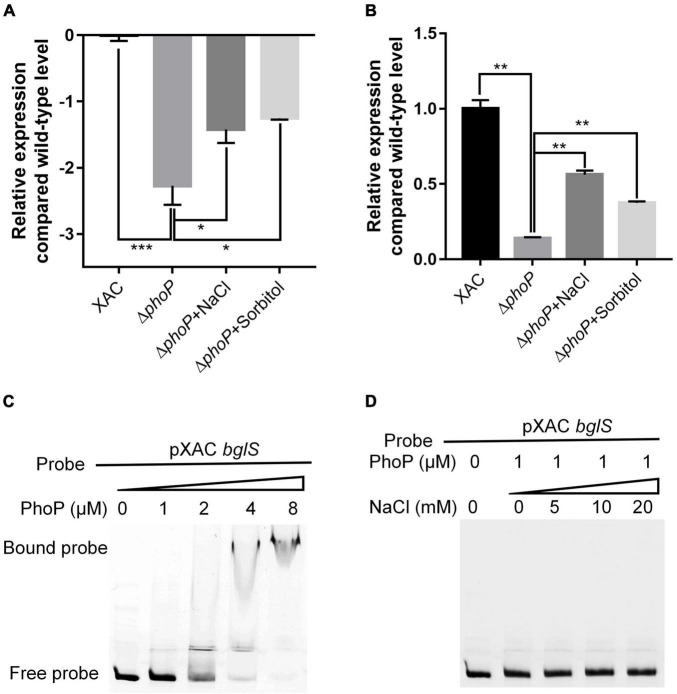
*X. citri* PhoP directly bound *bglS* promoters. **(A)** Relative expression of *bglS* as determined by RNA-Seq. **(B)** Relative expression of *bglS* as determined by quantitative reverse transcription PCR. **(C)** Gel shift assay showing that PhoP directly bound the promoters of *bglS*. **(D)** Gel shift assay showing the effect of 0.05 M NaCl on PhoP binding of *bglS* promoters. PhoP (0, 1, 2, 4, or 8 μM) were added to reaction mixtures containing 50 ng of probe DNA, and the reaction mixtures were separated on polyacrylamide gels. Error bars, means ± standard deviations (*n* = 3). *P < 0.05, **P < 0.01, ***P < 0.001, assessed by one-way ANOVA. Quantitative reverse transcription PCR and gel shift assay were repeated three times with similar results.

PhoP is a conserved global transcriptional regulator that is essential to produce virulence (Lee et al., 2008; Ren et al., 2016). RNA-Seq and RT-qPCR showed that *bglS* expression in Δ*phoP* mutant strain was compromised as compared to wild-type *X. citri* strain. It indicates that PhoP might regulate *bglS* expression by directly binding to its promoter region (Figures 6A,B).

To verify the *bglS* promoter for in-depth genetic analyses, a series of RT-PCR primers (Supplementary Table 2 and Supplementary Figure 8A) was designed to determine the intergenic transcripts crossing the adjacent genes. As shown in Supplementary Figure 8B, the successful amplification of corresponding intergenic transcripts revealed that *bglS* and XAC1449 likely constitute a single transcription unit suggesting that the *bglS* promoter is located upstream of XAC1449. Based on the identified PhoP-binding motif (Allenby et al., 2012), a potential binding site of *X. citri* PhoP was identified in the upstream region of XAC1449 and *bglS* operon: 5′-GATCACAGCAGGATCATG-3′ (Supplementary Figure 9).

Electrophoretic mobility gel shift assay (EMSA) was carried out to examine the direct binding of PhoP to *bglS* promoter. The PhoP protein was expressed in *E. coli* BL21 (DE3), and N-terminal-His 6-tagged versions of the proteins were purified with nickel chelate chromatography (Supplementary Figure 10). It was followed by the testing of PhoP ability to bind to the *bglS* promoter. A PCR-amplified 569 bp DNA fragment from the upstream of the XAC1449 translational start site (pXAC *bglS*) was used as a probe. The addition of purified PhoP protein, ranging from 0 to 8 μM, to the reaction mixtures (20 μL at 28°C for 25 min) caused a shift in the mobility of pXAC *bglS* DNA fragment. EMSA revealed strong PhoP binding with the pXAC *bglS* probe in a dose-dependent manner (Figure 6C). These results suggest that PhoP directly binds to the promoter region of *bglS* to regulate its transcription in *X. citri*. The effect of low osmostress to promote the expression of *bglS* was also compared with the *phoP* mutant strain (Figures 6A,B), but it could not enhance the PhoP ability to bind to the *bglS* promoter (Figure 6D). These results suggest that low osmostress induced BglS expression is independent of the PhoP regulatory pathway.

### Low Osmostress Induced β-Glucosidase Expression in *Xanthomonas citri*

**FIGURE 7:**
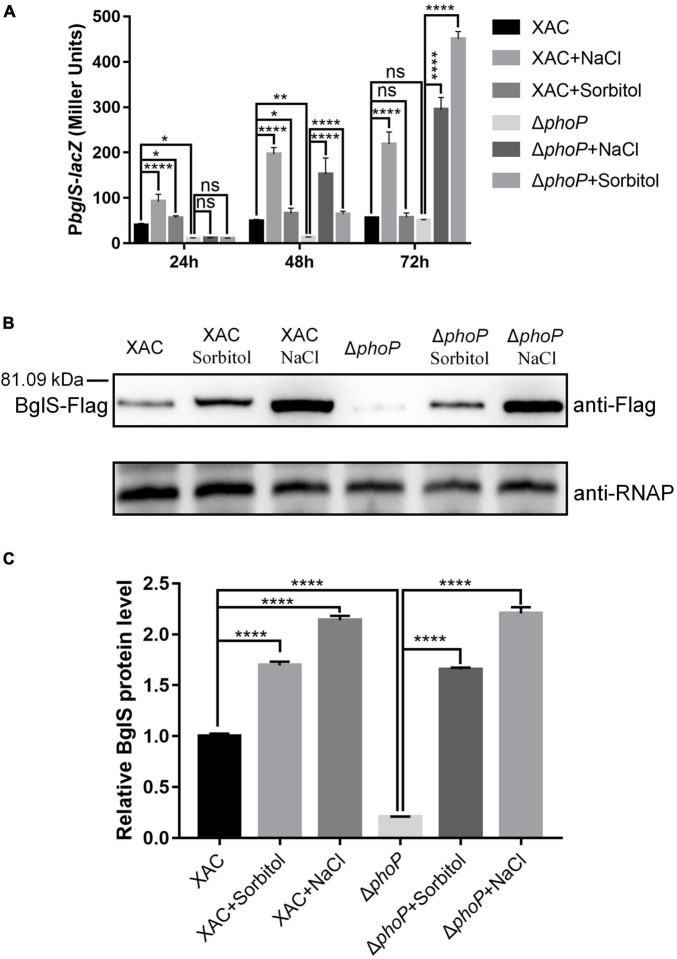
Low osmostress induced BglS expression in *X. citri*. **(A)** The effect of low osmostress on *bglS* gene expression was measured by assessing the β-galactosidase activity of the P*bglS*-lacZ transcriptional fusions in the *X. citri* and Δ*phoP* strains. **(B)** Expression of BglS-Flag in the *X. citri* and Δ*phoP* strains in NYG or NYG supplemented with 0.05 M NaCl or Sorbitol to an OD_600_ of 1.0. The Western blot was performed by using anti-Flag antibodies. The α subunit of RNA polymerase was used as an internal loading control and detected through a specific antibody (anti-RNAP). **(C)** Quantification and statistical analysis of BglS protein levels based on three biological replicates of the experiment mentioned in section **(B)**. Error bars, means ± standard deviations (*n* = 3). *P < 0.05, **P < 0.01, ****P < 0.0001, assessed by one-way ANOVA. All experiments were repeated three times with similar results.

Our results confirmed that low osmostress could promote the mRNA abundance of *bglS* as compared to *phoP* mutant strain (Figures 6A,B). A P*bglS*-lacZ reporter system was constructed in *phoP* mutant and wild-type strains to study the regulation by low osmotic stress. Consistent with the RNA-Seq and RT-qPCR results, deletion of *phoP* reduced *bglS* expression levels (Figure 7A). Importantly, osmotic pressure (NaCl and Sorbitol) at a low concentration of 0.05 M activated lacZ expression in the wild-type and *phoP* mutant strains (Figure 7A).

To further detect low osmostress induced *bglS* expression, a Flag tag-containing strain (XAC: *bglS*-Flag and Δ*phoP*: *bglS*-Flag) was constructed using homologous recombination methods (Supplementary Figure 11). This strain contained a Flag protein-coding sequence before the stop codon of *bglS* in the *X. citri* genome (Supplementary Figure 12). XAC: *bglS*-Flag and Δ*phoP*: *bglS*-Flag strains were cultured in NYG or NYG supplemented with 0.05 M NaCl or 0.05 M Sorbitol to OD_600_ of 1.0, and bacteria were collected for western blotting to detect the level of *bglS*-Flag. As shown in Figures 7B,C, the accumulation of BglS proteins was significantly decreased in the *phoP* mutant strain. Furthermore, the BglS protein levels rapidly increased in wild-type and *phoP* mutant strains treated with 0.05 M NaCl and 0.05 M sorbitol as compared to without osmostress treatment (Figures 7B,C). These results suggest that low osmostress was critical for inducing BglS expression and low osmostress induced BglS expression is probably independent of the PhoP regulatory pathway.

## Discussion

**FIGURE 8:**
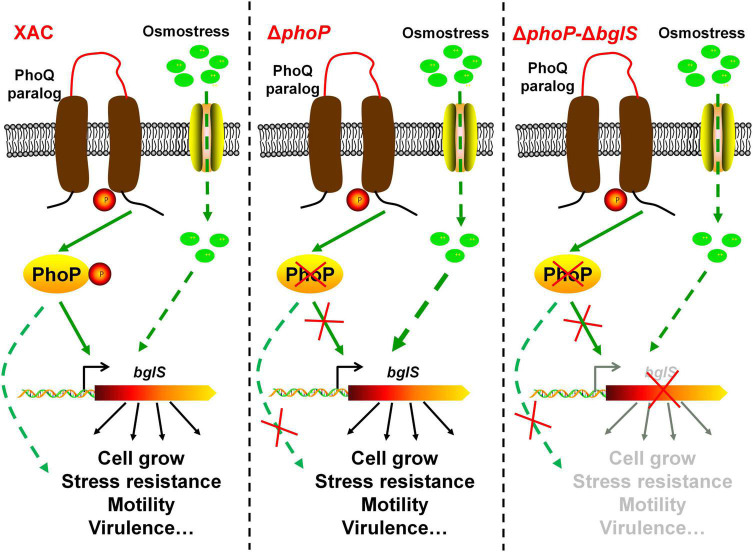
Model of speculation about PhoP and low osmostress regulation mechanism. The proposed regulatory pathways of PhoP and low osmostress based on our observations and previous studies. Two-component system response regulator PhoP is critical for *X. citri* growth and virulence. In the wild type, PhoP can directly regulate the expression of BglS. The deletion of *phoP* (Δ*phoP*) from *X. citri* significantly compromised the growth and virulence as compared to the wild-type strain. Simultaneously, the expression of *bglS* gene was significantly down-regulated in *phoP* mutant. This study revealed that the physiological deficiency of *phoP* mutant can be reversed by low osmostress induced β-glucosidase (BglS) expression and proposes a novel regulatory mechanism of β-glucosidase activation through low osmostress to reverse the fitness deficiency in *X. citri*.

The stress response is necessary for the organisms to adapt and survive in adverse environmental conditions (Dessaux et al., 2020). Bacterial cells are highly pressurized and contain a strongly crowded cytoplasm that is hyperosmotic to the environment (Bremer and Kramer, 2019; Schuster et al., 2020). To avoid dehydration in a hyperosmotic environment, most of the microorganisms modulate gene expression in response to specific high-concentration osmostress. However, the collective response of bacteria to low osmostress is still unknown. This study provides biochemical, genetic, and physiology evidence to demonstrate that low osmostress reverses fitness disadvantage by activating a novel β-glucosidase in *X. citri*. We also found that the deletion of *phoP* from *X. citri* significantly compromised the growth and virulence as compared to the wild-type strain. However, the physiological defects of *phoP* mutant were recovered at low osmostress by inducing BglS expression (Figure 8).

The PhoQ/PhoP two-component system is well known to respond to environmental changes including varying levels of magnesium, pH, AMPs, and osmolarity (Dalebroux et al., 2014; Yadavalli et al., 2016; Qadi et al., 2017; Yuan et al., 2017). PhoP, as a major regulator, controls the expression of approximately 2% of the genome including virulence gene and lipopolysaccharides (LPS) modifications (Guo et al., 1998; Dalebroux and Miller, 2014). *phoP*-*phoQ* of *X. campestris* pv. *campestris* are essential genes and can be genetically complemented by their orthologs from *P. aeruginosa* (Peng et al., 2017). However, a correct *phoP*_*Xcc*_ mutant could not be obtained without complementing *phoP*_*Xcc*_ gene. During this study, *phoP* mutant was successfully achieved in *X. citri* by deleting the *phoP* (XAC4023) gene, which is inconsistent with previous studies on *X. campestris* PhoP (Peng et al., 2017). *phoP* mutant was found to possess a significantly slower growth rate than the wild-type strain (Figure 1). We found that growth of *phoQ* deletion mutant on YEB and NYG medium was not affected compared with *X. citri* wild type (Figure 1). However, the molecular mechanism that deletion of *phoQ* do not affect the growth of *X. citri* still needs to be elucidated in the future.

The *phoP* deletion resulted in a severe growth reduction, and the low osmostress condition recovered the growth rate of the *phoP* mutant compared with wild type. To investigate the role of low osmostress in enhancing the growth of *phoP* mutant strain on NYG plates in *X. citri*, transcriptome analyses (RNA-Seq) were performed for the wild-type, *phoP* mutant, and *phoP* mutant supplemented with 0.05 M NaCl or 0.05 M sorbitol. PhoP and low osmostress co-regulation genes were screened in *X. citri*. RNA-Seq was performed to compare the transcriptional profiles of the wild-type, *phoP* mutant strain, and *phoP* mutant supplemented with 0.05 M NaCl or 0.05 M sorbitol. Two genes were found (Supplementary Figure 6) and annotated as XAC1448: β-glucosidase (BglS) and XAC2312: membrane protein (Supplementary Figure 6D).

Interestingly, plasmid-based overexpression of *bglS* significantly promoted the growth of *phoP* mutant strain in NYG plates (Figure 4A). Compared with the wild-type strain, the deletion of *bglS* damaged the growth of *phoP* mutant and low osmostress also could not promote the growth of Δ*phoP*-Δ*bglS* mutant on NYG plates (Figure 4B). However, the growth defect phenotypes of the Δ*phoP*-Δ*bglS* mutants were restored to the level of wild-type strain by introducing *bglS* into Δ*phoP*-Δ*bglS* (Figure 4B). These results suggest that BglS is critical for reversing the fitness disadvantage of *phoP* mutant. The role of low osmostress in inducing BglS expression to reverse the fitness disadvantage of *phoP* mutant was further confirmed by determining the protein levels of BglS in *X. citri* and *phoP* mutant strains in NYG medium or NYG supplemented with 0.05 M NaCl or 0.05 M sorbitol. The results revealed that the BglS protein levels rapidly increased in wild-type and *phoP* mutant strains treated with 0.05 M NaCl and 0.05 M sorbitol as compared to without osmostress treatment (Figure 7). However, Δ*bglS* mutant strain and complemented strains (Δ*bglS*/*bglS*) did not impair the growth of *X. citri* (Supplementary Figure 13) suggesting that BglS may only be used by *X. citri* for an emergency response to maintain the fitness.

The activation and inhibition of genes are often regulated by specific transcription factors in organisms (Xu et al., 2018; Li et al., 2020a,b). The data showed that although PhoP can positively regulate BglS expression, however, BglS expression can also be induced by low osmostress. The detailed reason for this interesting observation still requires further investigation. Some specific transcription factors might employ additional uncharacterized mechanisms to induce the BglS expression at low osmostress in *X. citri*. This hypothesis will be focused on during our future studies.

*X. citri* BglS has been predicted to possess two domains, Glycosyl hydrolase family 3 N terminal domain and Fibronectin type III-like domain, which are associated with enzymatic saccharification. The β-glucosidase are ubiquitous and play various biological roles due to their wide range of substrate specificities (Harnpicharnchai et al., 2009; Monteiro et al., 2020). β-glucosidases catalyze the hydrolysis of β-1,4, β-1,3, and β-1,6 glucosidic linkages from the non-reducing end of short-chain oligosaccharides, alkyl and aryl β-D-glucosides, and disaccharides (Kooloth Valappil et al., 2019; Monteiro et al., 2020). However, BglS role in recovering the *phoP* mutant growth is unknown. Elucidation of the detailed mechanism requires further investigation.

Based on the results, a schematic model was proposed in this study as shown in Figure 8. In short, these results not only provide novel molecular insights into multiple biological functions of PhoP in *X. citri*, but also present experimental evidence about the role of low osmostress in inducing BglS expression to reverse the *phoP* mutant fitness disadvantages including cell growth and virulence.

## Experimental Procedures

### Bacterial Strains, Plasmids, and Growth Conditions

Strains and plasmids used in this study are listed in Supplementary Table 1. *E. coli* strains were grown in Luria-Bertani medium (10 g L^–1^ tryptone, 5 g L^–1^ yeast extract, 10 g L^–1^ NaCl, pH 7.0) at 37°C. *X. citri* strains were grown at 28°C in NYG medium (5 g L^–1^ peptone, 3 g L^–1^ yeast extract, 20 g L^–1^ glycerol, pH 7.0) and YEB medium (10 g L^–1^ peptone, 5 g L^–1^ yeast extract, 10 g L^–1^ NaCl, 5 g L^–1^ sucrose, 0.5 g L^–1^ MgSO_4_ pH 7.5). Tryptone, peptone, beef extract, and yeast extract were purchased from Sangon Biotech (Shanghai, China) to prepare culture medium. Antibiotics such as sodium ampicillin (100 μg/mL), kanamycin sulfate (30 μg/mL), and gentamycin (30 μg/mL) were added for *E. coli* and *X. citri*, if required. Bacterial growth in a liquid medium was determined by measuring optical density at 600 nm (OD_600_) using a Bioscreen-C Automated Growth Curves Analysis System (Oy Growth Curves FP-1100-C, Helsinki, Finland).

### Gene Deletion and Complementation

The in-frame deletions in *X. citri* were generated *via* double-crossover homologous recombination (Li et al., 2017, 2020b) using the primers listed in Supplementary Table 2. The flanking regions of each gene were PCR-amplified and cloned into the suicide vector pK18mobsacB (Supplementary Table 1). The deletion constructs were transformed into the wild-type strain by electroporation, and kanamycin was used for the integration of the non-replicating plasmid into the recipient chromosome. A single-crossover integrant colony was spread on YEB medium without kanamycin and incubated at 28°C for 3 days. The culture was spread on YEB plates containing 15% sucrose after appropriate dilution. Colonies sensitive to gentamycin were screened by PCR using the primers listed in Supplementary Table 2, and the gene deletion strains were obtained.

DNA fragments containing the full-length genes along with their promoters were PCR amplified and cloned into the versatile plasmid pBBR1MCS5 (Kovach et al., 1995) to prepare gene complementation constructs. The resulting plasmids were transferred into the *X. citri* strain by electroporation, and the transformants were selected on LB plates containing gentamycin.

### Measurement of Extracellular Enzymatic Activity and Swimming Motility

Relative activities of extracellular enzymes were assayed as previously described (Wei et al., 2007; Yu et al., 2016). Two microliter of each *X. citri* strain culture (OD 600 ≈ 1.0) was spotted onto NYG agar plates containing 1% (w/v) skim milk (for protease), 0.5% (w/v) carboxymethylcellulose (for cellulase), or 0.1% (w/v) starch (for amylase) and incubated at 28°C for 24–48 h. Plates were stained where necessary according to Wei et al. (2007). Zones of clearance formed around the spot due to the degradation of the substrate were photographed. Three plates were inoculated in each experiment, and each experiment was repeated three times. The relative activity of the enzyme was indicated by the diameter of the clear zone.

Swimming motility was determined on semi-solid agar (0.3%). Bacteria were inoculated into the center of NYG plates containing 0.3% agarose. The plates were incubated at 28°C for 24 h before measuring the colony diameter.

### Pathogenicity and Hypersensitive Response Assays

HR and pathogenicity assays were performed as previously described (Andrade et al., 2014). Briefly, *X. citri* wild-type and *phoP* mutant strains were grown by shaking overnight at 28°C in YEB. The strains were then centrifuged, suspended in sterile water, and adjusted to a concentration of 10^8^ CFU/ml. Pathogenicity assays were conducted by infiltrating bacterial solutions of both strains (10^8^ CFU/ml) into the leaves with needleless syringes. Disease symptoms were photographed at 7 days post-inoculation. The strains were also tested for their ability to elicit an HR on *N. benthamiana* by infiltrating plant tissue with strains at 10^8^ CFU/ml with a needleless syringe. Plant responses were scored for HR in tobacco 5 days post-inoculation. Tobacco plants were grown in growth chambers at 25°C with a 12 h photoperiod. Experiments were repeated three times.

To detect enhanced *phoP* mutant virulence after low osmostress, the bacteria grown overnight in YEB medium were washed and re-suspended in sterile water supplemented with 0.05 M NaCl, and the concentrations were adjusted to 10^8^ CFU/ml. Pathogenicity and HR assays were detected as mentioned above.

### RNA-Seq

RNA-Seq assay was performed as previously described (Liao et al., 2019; Li et al., 2020b). Briefly, the wild-type, *phoP* mutant strain and *phoP* mutant supplemented with 0.05 M NaCl or 0.05 M sorbitol were grown in NYG medium, and their cells were collected when OD_600_ reached 1.0 based on their growth curve. The collected cells were used for RNA extraction by the TRIzol-based method (Life Technologies, CA, United States), and RNA degradation and contamination were monitored on 1% agarose gels. Then, clustering and sequencing were performed by Novogene (Beijing, China). To analyze the DEGs between the wild-type, *phoP* mutant strain and *phoP* mutant supplemented with 0.05 M NaCl or 0.05 M sorbitol, the gene expression levels were further normalized using the fragments per kilobase of transcript per million (FPKM) mapped reads method to eliminate the influence of different gene lengths and amount of sequencing data on the calculation of gene expression. The edgeR package^1^ was used to determine DEGs across samples with fold changes ≥ 2 and a false discovery rate adjusted *P* (*q*-value) < 0.05 whereas the expression of the wild type was set to the value of 1. DEGs were then subjected to enrichment analysis of GO functions and KEGG pathways, and *q*-values were corrected using < 0.05 as the threshold.

### Quantitative Real-Time PCR

Quantitative real-time PCR was carried out according to Li et al. (2020b,2021). Bacterial cells were collected at the cell optical density (OD_600_) of 1.0 in NYG or NYG supplemented with 0.05 M NaCl or 0.05 M sorbitol. Total RNA was extracted using a TRIzol-based method (Life Technologies, CA, United States). Different steps were carried out to ensure the RNA quality including (1) the degree of RNA degradation and potential contamination were monitored on 1% agarose gels; (2) RNA purity (OD260/OD280, OD260/OD230) was checked using a NanoPhotometer^®^ spectrophotometer (IMPLEN, CA, United States); and (3) RNA integrity was measured using a Bioanalyser 2100 (Agilent, Santa Clara, CA, United States). The primers used in this assay are listed in Supplementary Table 2. Each RNA sample (400 ng) was subjected to cDNA synthesis using TransScript^®^ All-in-One First-Strand cDNA Synthesis SuperMix for qPCR (One-Step gDNA Removal) Kit (TransGen Biotech, Beijing, China) according to the manufacturer’s instructions. qRT-PCR was performed using TransStart Top Green qPCR SuperMix (TransGen Biotech) on a QuatnStudio™ 6 Flex Real-Time PCR System (Applied Biosystems, Foster City, CA, United States) as follows: denaturation at 94°C for 30 s, followed by 40 cycles at 94°C for 5 s and 60°C for 34 s. Gene expression analyses were performed according to the 2^–ΔΔ*^CT^*^ method with 16S rRNA as the endogenous control, and the expression of the wild type was set to the value of 1. The experiments were performed three times and three replicates were examined in each run.

### Protein Expression and Purification

Protein expression and purification were performed according to Li et al. (2017). To clone XAC *phoP* gene, the genomic DNA extracted from strain *X. citri* was used for PCR amplification with *Pfu* DNA polymerase and primers (Supplementary Table 2). PCR products were inserted into pET-28b (+) to produce plasmids pET-*phoP*. The *phoP* gene was confirmed through nucleotide sequencing by Genscript (Nanjing, Jiangsu, China). *phoP* with a vector-encoded His_6_-tagged N-terminus was expressed in *E. coli* BL21 (DE3), and purified with Ni-NTA agarose (Qiagen, Chatsworth, CA, United States) using a nickel-ion affinity column (Qiagen). Protein purity was monitored by SDS-PAGE.

### Electrophoretic Mobility Gel Shift Assays

Electrophoretic mobility gel shift assays (EMSA) were performed as described by Li et al. (2020b). DNA fragments including XAC *bglS* (569 bp) promoter region were used as probes in PhoP gel shift assays. The probe DNA (100 ng) was mixed with protein in a 20 μl reaction mixture containing 10 mM Tris (pH 7.5), 50 mM KCl, 1 mM dithiothreitol, and 0.4% glycerol. After incubation for 25 min at 28°C, the samples were electrophoresed on a 5% non-denaturing acrylamide gel in 0.5X TBE buffer at 4°C. The gel was soaked in 10,000-fold-diluted SYBR Green I nucleic acid Dye (Shanghai Sangon Biotech, China), and DNA was visualized at 300 nm.

### Western Blot Analysis

Western blot analysis was performed according to a standard laboratory protocol with minor modifications (Wang et al., 2018). The FLAG tag-containing strains (XAC: *bglS*-Flag and Δ*phoP*: *bglS*-Flag) were cultured in NYG or NYG supplemented with 0.05 M NaCl or 0.05 M sorbitol. Bacterial cells were collected at an OD_600_ of 1.5 to extract the total proteins, which were further separated by SDS-PAGE (12%) and immobilized onto polyvinylidene difluoride (PVDF) membranes using a semi-dry blot machine (Bio-Rad). The membranes were probed with a monoclonal antibody specific for the Flag-tag (1:5,000; Abmart), followed by the detection with horseradish peroxidase (HRP)-conjugated anti-rabbit secondary antibody (Abmart, Shanghai, China). RNA polymerase α subunit was used as a control for sample loading and antibody recognition. RNA polymerase α subunit was provided by Dr. Wei Qian (Chinese Academy of Sciences) (Deng et al., 2014).

### Construction of Reporter Strains and β-Galactosidase Measurement Assays

The P*bglS*-lacZ reporter was introduced into the *X. citri* wild type and *phoP* mutant strains by electroporation. The transconjugants were selected on YEB agar plates supplemented with tetracycline, and X-Gal. To measure of β-galactosidase activities, the overnight cultured bacteria were diluted to the same cell densities (OD 600 ≈ 0.01) in NYG medium or NYG supplemented with 0.05 M NaCl or 0.05 M sorbitol. The inoculated cultures were incubated at 28°C and 180 rpm. The cells were harvested to assess β-galactosidase activities according to Wang et al. (2019, 2021).

### Statistical Analyses

Experimental datasets were subjected to Analysis of variance using Graphpad prism 7.0. Significant effects of the treatment were determined by *F*-value (*P* = 0.05), whereas the separation of means was accomplished by Fisher’s protected least significant difference at *P* ≤ 0.05.

## Data Availability Statement

RNA-sequencing raw data are deposited into the NCBI’s Sequence Read Archive (SRA) and are accessible through BioProject series accession number PRJNA778446. The data that support the findings of this study are available from the corresponding author upon reasonable request.

## Author Contributions

KL and CC conceived and designed the experiments. KL, JL, MW, SQ, WW, and YZ carried out the experiments. KL, CC, and QL analyzed the data and prepared the figures. KL wrote the manuscript. CC, FL, and HW reviewed and revised the manuscript. All authors reviewed the manuscript.

## Conflict of Interest

The authors declare that the research was conducted in the absence of any commercial or financial relationships that could be construed as a potential conflict of interest.

## Publisher’s Note

All claims expressed in this article are solely those of the authors and do not necessarily represent those of their affiliated organizations, or those of the publisher, the editors and the reviewers. Any product that may be evaluated in this article, or claim that may be made by its manufacturer, is not guaranteed or endorsed by the publisher.

## Footnotes

1
http://www.r-project.org/
