# Supplementary material for: The Xanthomonas citri Reverse Fitness Deficiency by Activating a Novel β-Glucosidase Under Low Osmostress
Source: Front Microbiol. 2022 May 2;13:887967. doi: 10.3389/fmicb.2022.887967 (PMC9108719; doi:10.3389/fmicb.2022.887967)
Supplement: Supplementary file 1 [file Data_Sheet_1.PDF]

Supplementary Information

A phytopathogenic bacteria reverse fitness deficiency by activating a novel β-glucosidase under low osmotic stress

Kaihuai Li[1,3,#], Jinxing Liao[1,#], Ming Wei[1,2], Shanxu Qiu[1], Yancun Zhao[3], Haihong Wang[4], Qiongguang Liu[2], Fengquan Liu[3,*], Changqing Chang[1,2,*]

Contents

Fig. S1

Fig. S2

Fig. S3

Fig. S4

Fig. S5

Fig. S6

Fig. S7

Fig. S8

Fig. S9

Fig. S10

Fig. S11

Fig. S12

Fig. S13

Table S1

Table S2

Table S3

Table S4

Table S5

Fig. S1

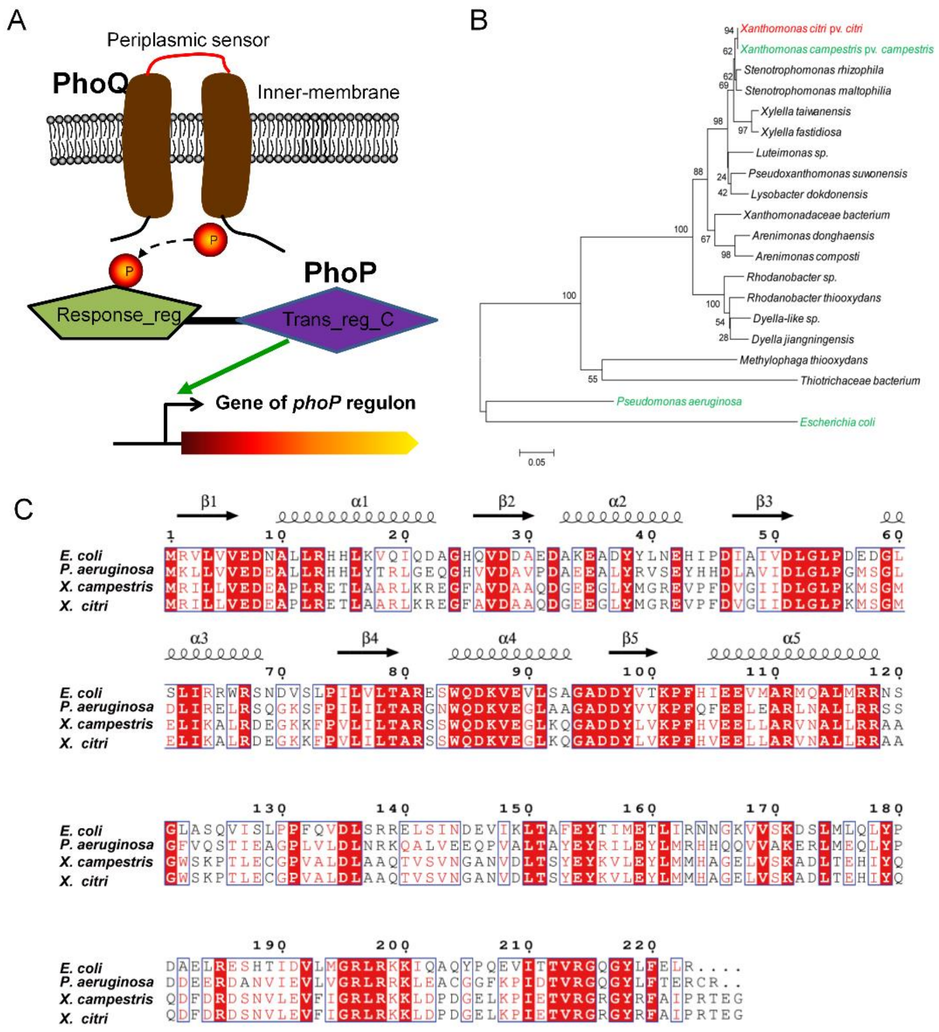

Fig. S1 Phylogeny and signal transduction of the PhoQ/PhoP two-component system, and alignment of *X. citri* PhoP with PhoP of known structure. **(A)** Schematic view of the PhoP and PhoQ secondary structures and cellular locations. 'P' in red circles represents the phosphoryl group that is transferred from PhoQ to PhoP. **(B)** Phylogenetic relationships of PhoP. Orthologous protein sequences from representative species of α-, β- and γ-Proteobacteria were used to construct the trees by the Neighbour-Joining method. The scale bar indicates the nucleotide substitutions per site. Phylogenetic trees were evaluated by bootstrapping (500 duplicates). Bacteria belonging to *Xanthomonas citri* are listed in red and *Xanthomonas campestris*, *Pseudomonas aeruginosa*, and *Escherichia coli* are listed in green. **(C)** Alignment of *X. citri*, *X. campestris*, *E. coli* and *P. aeruginosa* PhoP. Alignment was constructed with Clustal W based on identical residues.

Fig. S2

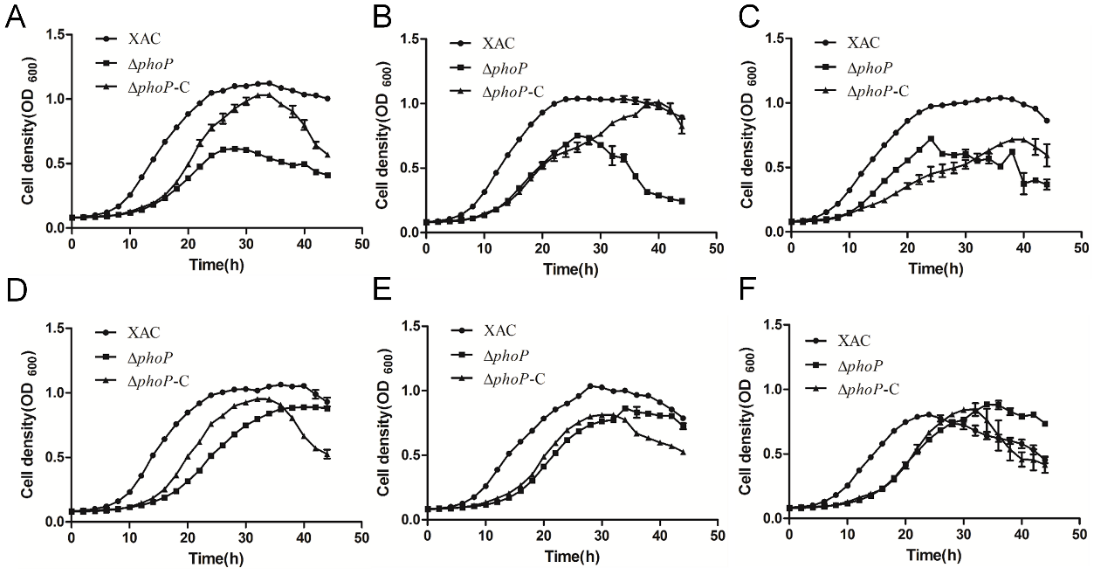

Fig. S2 Enhanced growth of *phoP* mutant under low-concentration osmostress. (A-C) Growth curves of bacterial strains in rich NYG medium supplemented with 0.02 M KCl, 0.05 M KCl, or 0.1 M KCl, respectively. **(D-F)** Growth curves of bacterial strains in rich NYG medium supplemented with 0.02 M NH_4Cl, 0.05 M NH_4Cl, or 0.1 M NH_4Cl, respectively. OD_{600} of cultures was monitored using a Bioscreen-C Automated Growth Curves Analysis System (Oy Growth Curves FP-1100-C, Helsinki, Finland). Error bars mean ± standard deviation (n = 3). All experiments were repeated three times with similar results.

Fig. S3

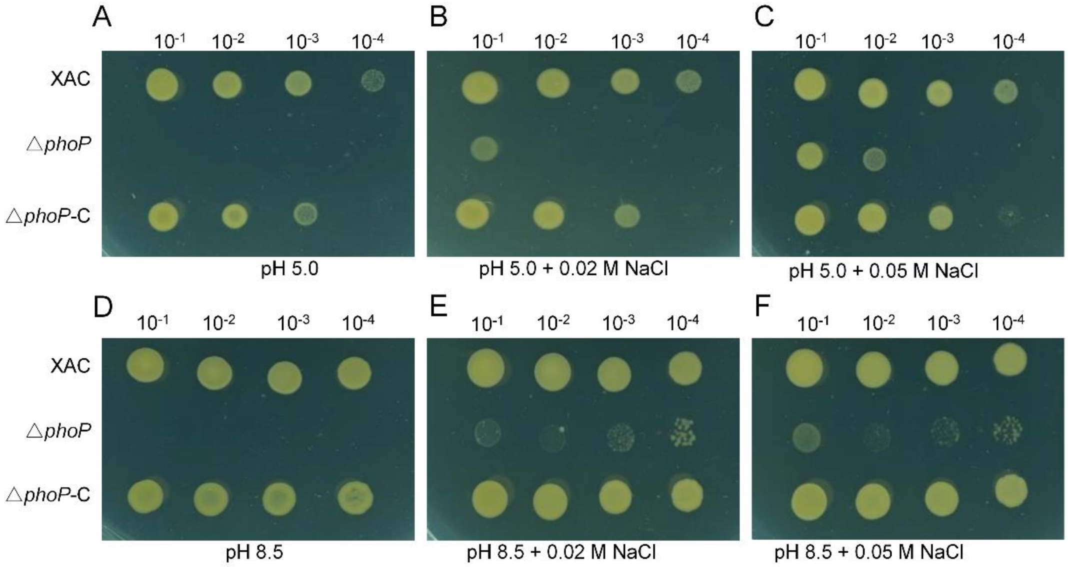

Fig. S3 Low osmostress promoted *phoP* mutant growth when *X. citri* was subjected to various environmental stresses. (A) Growth of bacterial strains on rich NYG medium plates supplemented with pH 5.0. **(B)** Growth of bacterial strains on rich NYG medium plates supplemented with pH 5.0 and 0.02 M NaCl. **(C)** Growth of bacterial strains on rich NYG medium plates supplemented with pH 5.0 and 0.05 M NaCl. **(D)** Growth of bacterial strains on rich NYG medium plates supplemented with pH 8.5. **(E)** Growth of bacterial strains on rich NYG medium plates supplemented with pH 8.5 and 0.02 M NaCl. **(F)** Growth of bacterial strains on rich NYG medium plates supplemented with pH 8.5 and 0.05 M NaCl. OD_{600} of cultures was monitored using a Bioscreen-C Automated Growth Curves Analysis System (Oy Growth Curves FP-1100-C, Helsinki, Finland). Error bars mean ± standard deviation (n = 3). All experiments were repeated three times with similar results.

Fig. S4

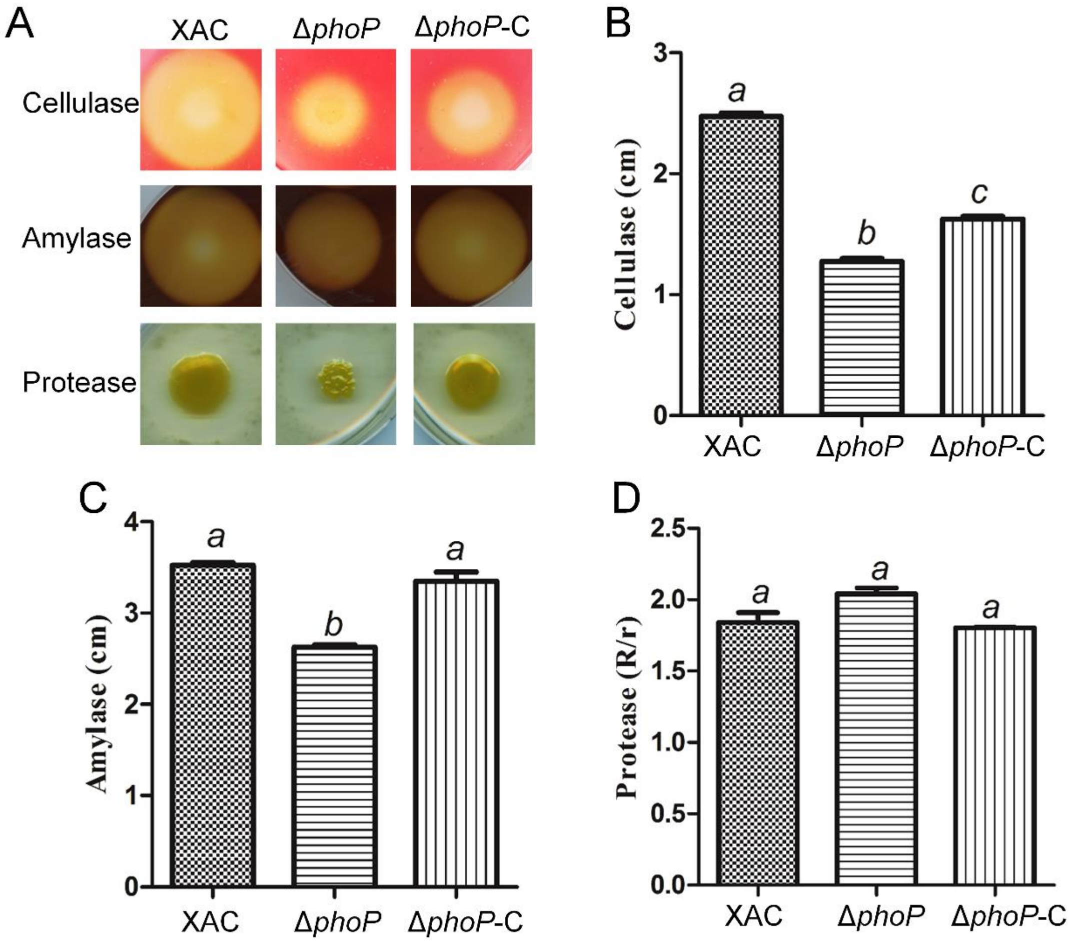

Fig. S4 Relative activity of extracellular enzymes produced by *X. citri* strains in NYG. Error bars, mean ± standard deviation (n = 3). All experiments were repeated three times with similar results.

Fig. S5

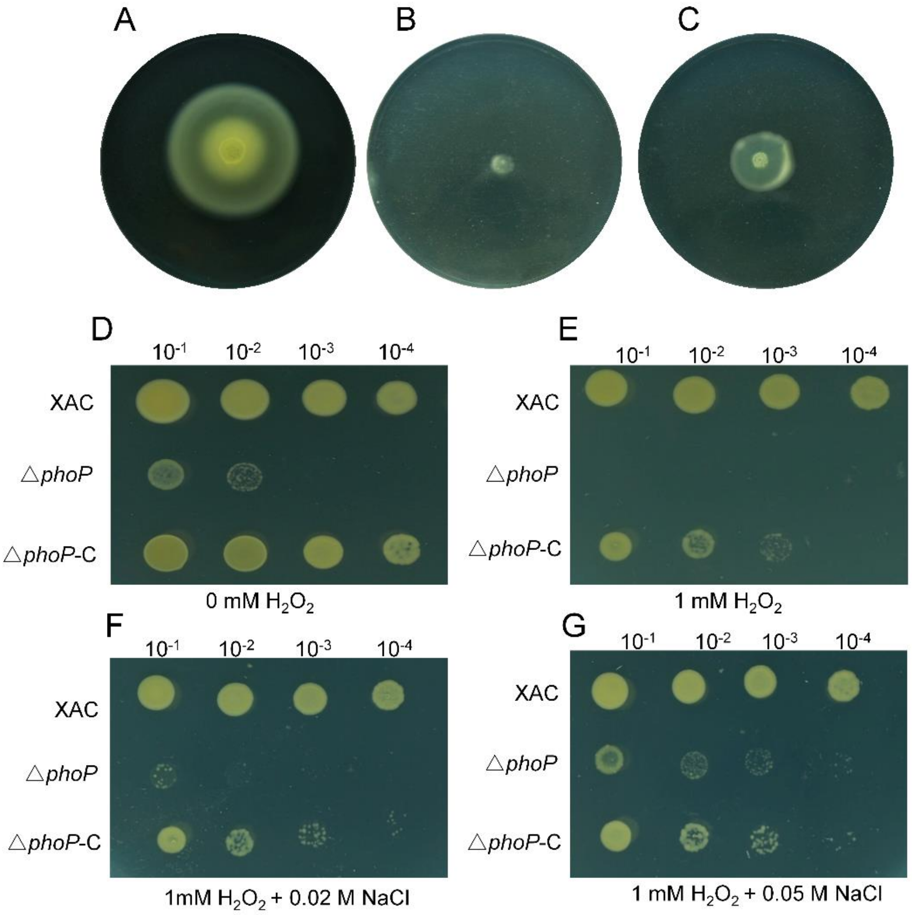

Fig. S5 *phoP* mutant affected *X. citri* swimming motility and response to oxidative stress. **(A)** Swimming motility of *X. citri* wild-type strain on rich NYG medium plates. **(B)** Swimming motility of *phoP* mutant strain on rich NYG medium plates. **(C)** Swimming motility of *phoP* mutant strain on rich NYG medium plates supplemented with 0.05 M NaCl. **(D)** Growth of bacterial strains on rich NYG medium plates at 28°C. **(E)** Response of *X. citri* wild-type strain against oxidative stress on rich NYG medium plates supplemented with H_2O_2 at final concentrations of 1mM at 28°C. **(F)** Response of *phoP* mutant strain against oxidative stress on rich NYG medium plates supplemented with H_2O_2 at final concentrations of 1mM at 28°C. **(G)** Response of *phoP* mutant strain against oxidative stress on rich NYG medium plates supplemented with 1mM H_2O_2 and 0.05 M NaCl at 28°C. All experiments were repeated three times with similar results.

Fig. S6

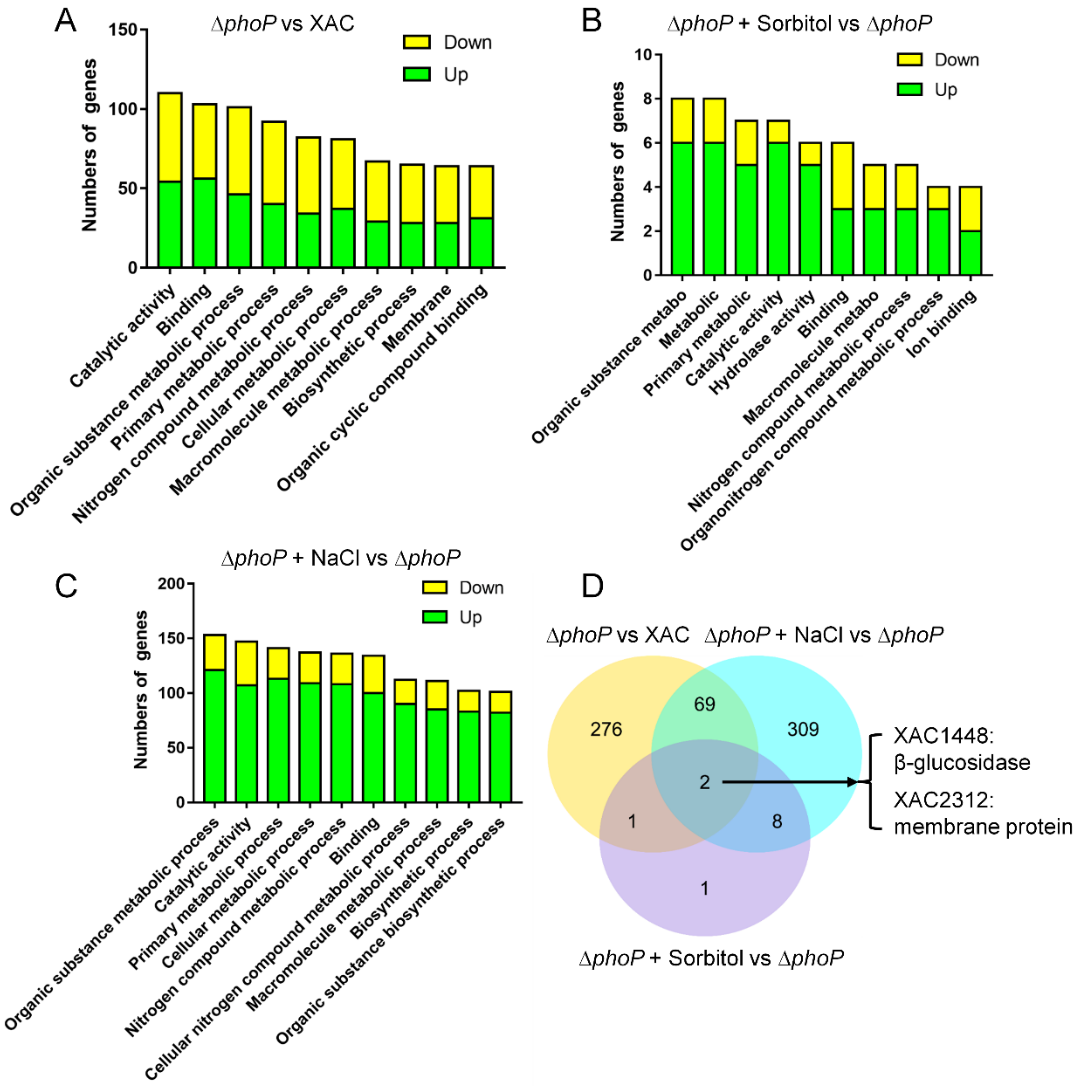

Fig. S6 Differential gene expression profiles between *X. citri* wild-type strain, *phoP* mutant and *phoP* mutant supplemented with 0.05 M NaCl or 0.05 M sorbitol as measured by RNA-seq (Log2 fold change ≥ 1) (A-C) GO term enrichment analysis of differentially expressed genes: Δ*phoP* vs XAC, Δ*phoP* vs Δ*phoP* + NaCl and Δ*phoP* vs Δ*phoP* + sorbitol, respectively. **(D)** Venn diagrams showing the gene overlapping on different mutant backgrounds.

Fig. S7

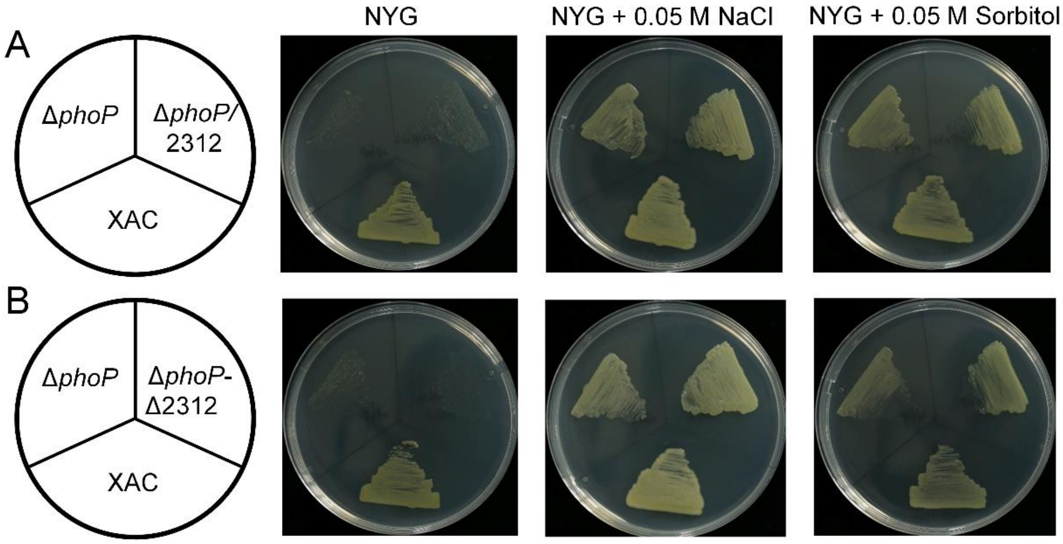

Fig. S7 Inactivation of XAC 2312 did not significantly affect the reversal of growth disadvantage of *phoP* mutant. (A-B) Growth of bacterial strains on rich NYG medium plates or NYG medium plates supplemented with 0.05 M NaCl or 0.05 M sorbitol.

Fig. S8

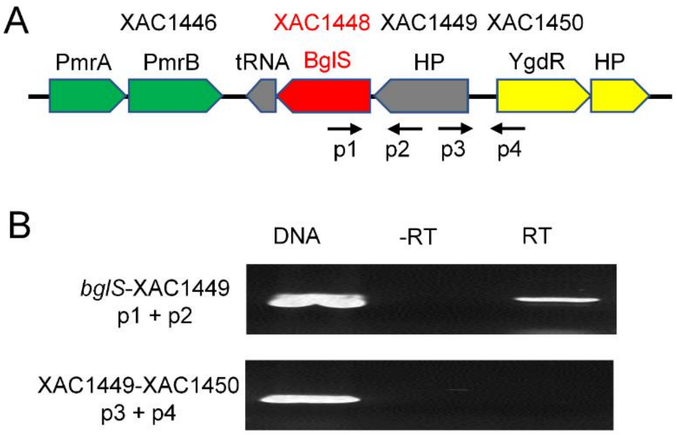

Fig. S8 Location of XAC1449 and *bglS* (XAC1448) in an operon. (A) Genomic localization of XAC1449 *and bglS* (XAC1448). Arrows indicate open reading frame regions of the genes and their transcriptional directions. Gene names are listed above whereas the primers used to verify operon structures by RT-PCR are indicated below. The primers are listed in Table S2. PmrA: multidrug resistance efflux pump; PmrB: multidrug resistance membrane translocase; Trna: tRNA-Leu; BglS: beta-glucosidase; HP: conserved hypothetical protein; YgdR: oligopeptide transporter. **(B)** Verification of operon organization by RT-PCR. The cDNA was reverse-transcribed with random primers using total RNA from *X. citri* grown in YEB medium at 28°C until the OD_{600} reached 1.0. PCR fragment lengths are listed on the right. RT represents amplification using cDNA transcribed from RNA as a template; -RT represents the negative control in which reverse transcriptase was absent during cDNA synthesis; DNA represents the positive control using DNA as the PCR template.

Fig. S9

bglS promoter

CCTGCAGCAACAGCGAGGTGATCAGGAACTGCACCAGGATGTTGCGCATG
CCGTAGAAGCTGAAACGCTCGCAGGCCTCGTTGCCGATGATGAAAGGAAT
CTGGAGCGGCAGCCGCGCGCCGGCAGATGCGCCTGGCGAGATGGAGGTG
GTCAAAGGCAGTCCGGTCATGCTGAAAACAGGCCCTATCGTACCGGCGCC
CACCCGCCGGCCGCCATCGCCCGCCCGGGAGTGCCGGACGCGGGCACCGA
GCGGATGCGCCTGGCGGACCTCACTTCGGCATGATCGATGACCTACAGGC
CGGCCGGAAACGACATTGCTCCAAGCGTTGCACAGCCTCCGCGTCGTTTG
CGCCTACCTCGAATGTCGCTCAGCTGGCCAACGAAAACCGGCCCCGCGTC

PhoP binding box

CCAGGTGAAACCCACGCACATGGCACCGATCACAGCAGGATCATGACGC

-35 box -10 box

AGTGCAGATTGGCCTTGCACGCTGGCTCGGCTACATTGCAAACGTTTTCCT
GGAACACTGGACCGCTGGATGccccgcttcactcgcctggccctgagcctgttgctgctga

Start codon

Fig. S9

Schematic illustration of transcription promoter regions of XAC1449 and *bglS* operon.

The PhoP-binding box, -10/-35 consensus boxes and XAC1449 start codon are marked by underline.

Fig. S10

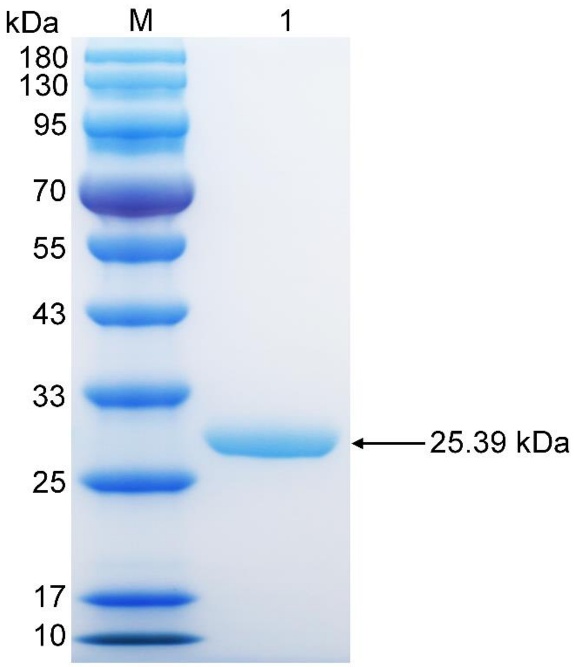

Fig. S10 SDS-PAGE (12%) analysis of purified PhoP cytoplasmic fragments. Lane M, molecular mass markers; lane 1, PhoP-His protein.

Fig. S11

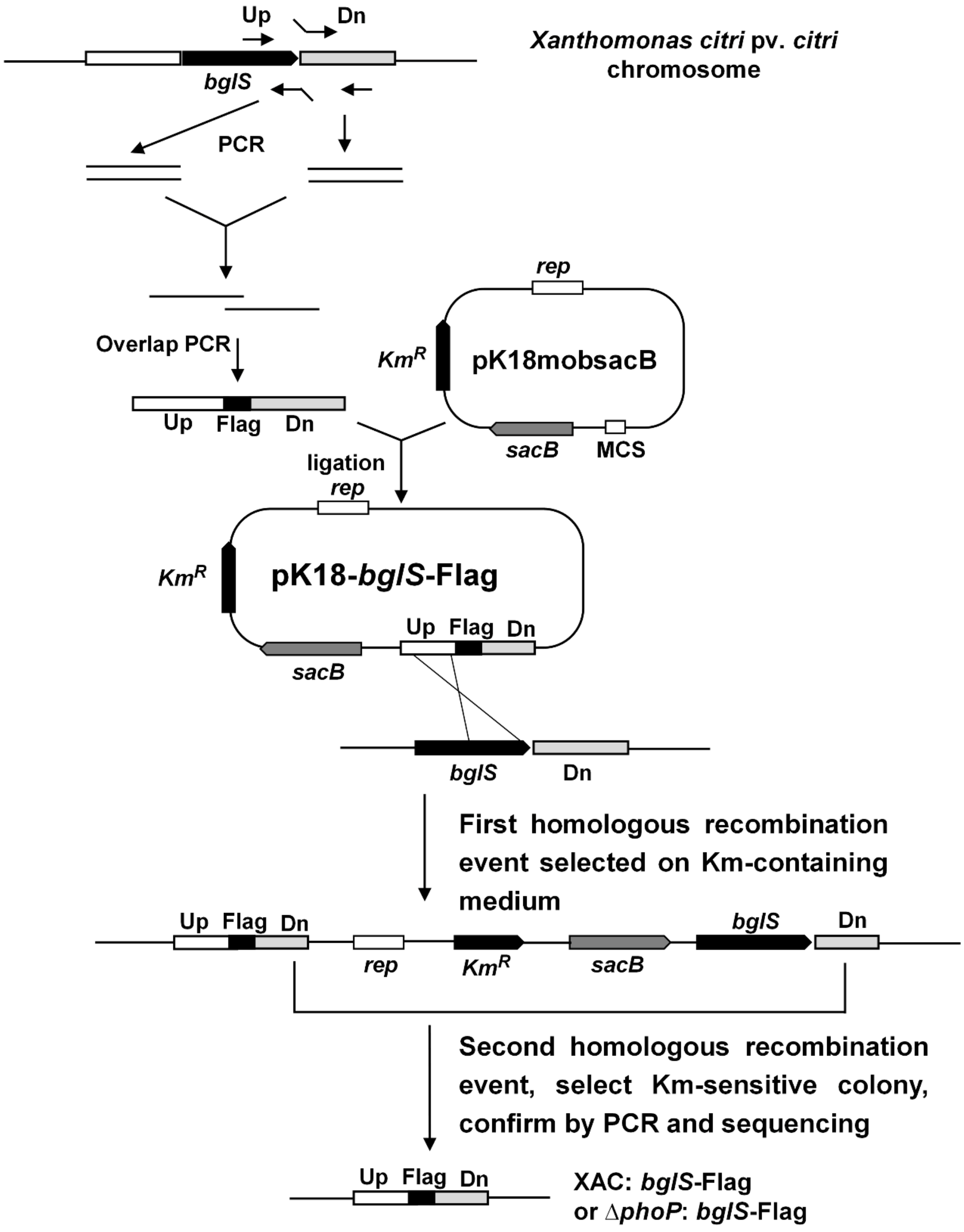

Fig. S11 BglS-Flag fusion protein knock-in scheme. DNA fragments containing upstream (labeled up) and downstream (labeled down) regions of the insertion region and overlap region (FLAG tag sequence) were used as the 5′ and 3′ fragments for homologous recombination. Additional details are shown in the experimental procedures.

Fig. S12

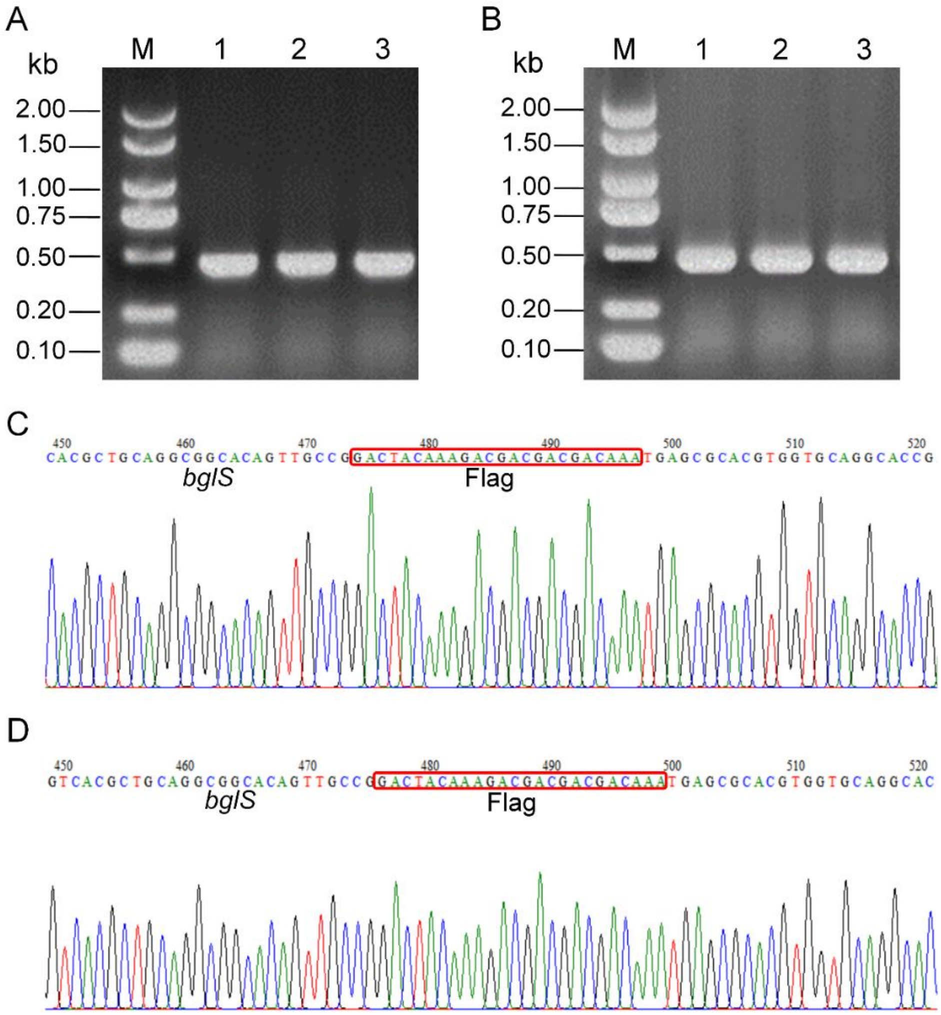

Fig. S12 PCR and sequencing analysis of Flag knock-in strains. (A) PCR detecting the *bglS* downstream (labeled down) regions and overlap region (Flag-tag) sequence in *X. citri* strain. M, DNA Marker; lane 1-3, XAC: *bglS*-Flag. **(B)** PCR detecting the *bglS* downstream (labeled down) regions and overlap region (Flag-tag) sequence in *phoP* mutant strain. M, DNA Marker; lane 1-3, Δ*phoP*: *bglS*-Flag. **(C)** Sequencing analysis of Flag sequences in *X. citri* knock-in strains. **(D)** Sequencing analysis of Flag sequences in *phoP* mutant knock-in strains. The primers used in this assay are listed in Table S2.

Fig. S13

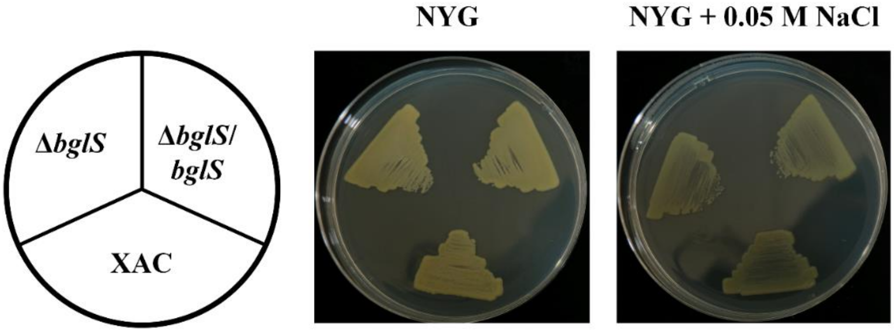

Fig. S13 Inactivation of *bglS* did not significantly affect *X. citri* growth. Growth of bacterial strains on rich NYG medium plates or NYG medium plates supplemented with 0.05 M NaCl.

Table S1. Bacterial strains and plasmids used in this study

Strains	Relevant characteristics	References
E.coli		
**BL21(DE3)**	F^- *dcm omp T hsdS*($r_B^- m_B^-$) *gal* (λDE3)	Lab collection
**DH5α**	F^- *deoR endA1 gyrA96 hsdR17*($r_K^- m_K^+$) *recA1 relA1 supE*44 *thi-1* Δ(*lacZYA-argF*)*U*169(φ80*lacZ*ΔM15)	Lab collection
X. citri* pv.*citri		
**XAC XHG3**	Wild-type strain	
**Δ*phoP***	The *phoP* in-frame deletion mutant of strain XAC XHG3	This study
**Δ*phoP*-C**	Gm^r, the *phoP* in-frame deletion mutant harboring the *phoP* expression cosmid pBBR1-*phoP*.	This study
**Δ*bglS***	The *bglS* in-frame deletion mutant of strain XAC XHG3	This study
**Δ*bglS*/*bglS***	Gm^r, the *bglS* in-frame deletion mutant harboring the *bglS* expression cosmid pBBR1-*bglS*.	This study
**Δ*phoP*-Δ*bglS***	The *phoP* and *bglS* in-frame deletion mutant of strain XAC XHG3	This study
**Δ*phoP*-Δ*bglS*/*bglS***	Gm^r, the *phoP* and *bglS* in-frame deletion mutant harboring the *bglS* expression cosmid pBBR1-*bglS*.	This study
**Δ*phoP*/*bglS***	Gm^r, the *phoP* in-frame deletion mutant harboring the *bglS* expression cosmid pBBR1-*bglS*.	This study
**Δ*phoP*-Δ2312**	The *phoP* and XAC2312 in-frame deletion mutant of strain XAC XHG3	This study

**Δ*phoP*/XAC2312**	Gm^r, the *phoP* in-frame deletion mutant harboring the XAC2312 expression cosmid pBBR1-XAC2312.	This study
**Δ*phoP*-Δ2312/ XAC2312**	Gm^r, the *phoP* and XAC2312 in-frame deletion mutant harboring the XAC2312 expression cosmid pBBR1-XAC2312.	This study
**XAC (*bglS*-Flag)**	XAC strain expressing Flag-fused *bglS* at the genome level	This study
**Δ*phoP* (*bglS*-Flag)**	Δ*phoP* strain expressing Flag-fused *bglS* at the genome level	This study
**Plasmids**		
**pK18mobsacB**	Km^r, *sacB*-based gene replacement vector	(Schafer et al., 1994)
**pBBR1MCS5**	Gm^r, Broad host range cloning vector.	(Kovach et al., 1995)
**pBBR1-*phoP***	Gm^r, XAC *phoP* in pBBR1MCS5	This study
**pBBR1-*bglS***	Gm^r, XAC *bglS* in pBBR1MCS5	This study
**pBBR1-2312**	Gm^r, XAC2312 in pBBR1MCS5	This study
**pK18-Δ*phoP***	Km^r, XAC *phoP* in-fame deletion fragment inserted to pK18mobsacB vector between *Eco*RI/*Hin*dIII sites	This study
**pK18-Δ*bglS***	Km^r, XAC *bglS* in-fame deletion fragment inserted to pK18mobsacB vector between *Eco*RI/*Xba*I sites	This study
**pK18-Δ2312**	Km^r, XAC2312 in-fame deletion fragment inserted to pK18mobsacB vector between *Eco*RI/*Xba*I sites	This study
**pME2-P*bglS***	pME2-lacZ containing promoter of *bglS*	
**pME2-P2312**	pME2-lacZ containing promoter of XAC2312	

Km^R, Gm^R, Rif^R: Kanamycin, Gentamicin, Rifampicin resistance, respectively.

Table S2. Sequences of the PCR primers used in this work

Primer name	Primer sequence (5' to 3')	Digestion sites [a]
**For deletion**		
phoP EcoRI	aattGAATTCtccgatgctcgaccttcaat	*Eco*RI
phoP up1	cggaatggcgaagcgataaccgaccaaaaggatacgcatg	
phoP down1	catgcgtatccttttggtcggttatcgcttcgccattccg	
*phoP Hin*dIII	tataAAGCTTcgcaccacttccacatacag	*Hin*dIII
*bglS Eco*RI	accgGAATTCatcgtgggatgagcgctac	*Eco*RI
bglS up1	aattGGTACCttcgggcttgggaatctt	*Kpn*I
bglS down1	aattGGTACCcagctctacgtcgccctg	*Kpn*I
*bglS Xba*I	ctagGAATTCtcgaacctccacgaagttg	*Xba*I
2312 *Eco*RI	accgGAATTCcatgacccgactgatgcg	*Eco*RI
2312 up1	aattGGTACCagattggtggcggtgacct	*Kpn*I
2312 down1	aattGGTACCtaccggaacattgcaggact	*Kpn*I
2312 *Xba*I	ctagTCTAGAttgacctcgttggtggtgtc	*Xba*I
**For XAC (*bglS*-Flag) or Δ*phoP* (*bglS*-Flag) strains construction**		
bglS-Flag P1	accgGAATTCgtcggttacaagtggttcgc	*Eco*RI
bglS-Flag P2	cctgcaccacgtgcgcTCATTTGTCGTCGTCGTCTTTGTAGTCCGGCAACTGTGCCGCCTGCA	
bglS-Flag P3	TGCAGGCGGCACAGTTGCCGGACTACAAAGACGACGACGACAAATGAgcgcacgtggtgcagg	
bglS-Flag P4	ctagGAATTCatcttcttcctgtggttggc	*Xba*I
**For in trans expression**		
phoP-F	ccAAGCTTgggagagtgcatgcgtatcc	*Hin*dIII
phoP-R	cgGGATCCacttggtcttcctgctcgtt	*Bam*HI
phoP Res P1	accgGAATTCatgcgtatccttttggtc	*Eco*RI

phoP Res P2	cataTCTAGAattgacgcgcgccagcaac	*Xba* I
phoP Tran P1	atcgGAATTCatggccaacgtcgatctgaccag	*Eco*RI
phoP Tran P2	ctagTCTAGAaatggcgaagcgataaccg	*Xba* I
P*bglS* F	cccAAGCTTtccaacgaaagccatcagac	*Hin*dIII
P*bglS* R	ccgCTCGAGgacgattctgatgggctgta	*Xho* I
P2312 F	cccAAGCTTtgatttcctgcagcaacag	*Hin*dIII
P2312 R	ccgCTCGAGaagtcagcagcaacaggctc	*Xho* I
**For RT-PCR**		
RT-*bglS* F	ACTCAACGACATGGAAACGC	
RT-*bglS* R	CGCAACCGTAGATACCGTTG	
RT-16S rRNA F	tcccggtgtagcagtgaaat	
RT-16S rRNA R	ccagttcgcatcgtttaggg	

[a] Underlined, restriction endonuclease sites.

Table S3. List of genes differentially expressed in the *phoP* mutant compared to the wild-type strain (Log2 fold change ≥ 1).

Gene ID	Gene name	Gene function	Log_2 fold change
XAC_RS00090	XAC0017	radical SAM protein	-2.3742
XAC_RS00120	XAC0023	PDZ domain-containing protein	-1.1886
XAC_RS00140	XAC0027	PepSY domain-containing protein	1.452
XAC_RS00160	XAC0030	cellulase	-2.2516
XAC_RS00165	XAC0031	NAD(P)-dependent alcohol dehydrogenase	-1.418
XAC_RS00335	XAC0065	phage tail protein	1.3341
XAC_RS00375	XAC0072	saccharopine dehydrogenase	-1.2128
XAC_RS00405	XAC0078	DUF1998 domain-containing protein	-1.5097
XAC_RS24900	XAC0096	hypothetical protein	2.9976
XAC_RS00495	XAC0097	hypothetical protein	3.2
XAC_RS22770	XAC0098	hypothetical protein	3.8298
XAC_RS00555	XAC0108	host attachment protein	1.5627
XAC_RS00580	XAC0112	EcsC family protein	1.404
XAC_RS00725	XAC0139	hypothetical protein	1.6505
XAC_RS00765	XAC0146	hypothetical protein	1.4604
XAC_RS00775	XAC0147	hypothetical protein	1.7566
XAC_RS00790	XAC0150	hypothetical protein	1.6843
XAC_RS00915	XAC0176	TonB-dependent siderophore receptor	4.4129
XAC_RS01125	XAC0215	hypothetical protein	-1.501
XAC_RS01135	XAC0217	glycosyl transferase	-2.7662
XAC_RS01165	XAC0223	Ax21 family protein	-5.3925
XAC_RS01175	XAC0225	sensor histidine kinase	-2.1967
XAC_RS01205	XAC0231	hypothetical protein	1.3947
XAC_RS01210	XAC0232	DUF4156 domain-containing protein	-1.8522
XAC_RS01390	XAC0267	hypothetical protein	-1.2691
XAC_RS01400	XAC0269	DUF3325 domain-containing protein	2.8633
XAC_RS01405	XAC0270	PepSY domain-containing protein	3.3372
XAC_RS01415	XAC0272	DUF4198 domain-containing protein	4.1439
XAC_RS01645	XAC0314	hypothetical protein	-2.4077
XAC_RS01995	XAC0378	hypothetical protein	-2.1291
XAC_RS02175	XAC0415	EscC/YscC/HrcC family type III secretion system outer membrane ring protein	-1.3106
XAC_RS02205	XAC0421	phosphoglycerol transferase I	-1.2369
XAC_RS22880	XAC0468	hypothetical protein	1.8498
XAC_RS02480	XAC0476	anthranilate synthase component I	-1.2777
XAC_RS02560	XAC0492	bacterioferritin	2.7173
XAC_RS02630	XAC0506	membrane protein	1.4064
XAC_RS02645	XAC0508	LysR family transcriptional regulator	-1.494

XAC_RS02740	XAC0525	hypothetical protein	3.1609
XAC_RS02815	XAC0540	ribonuclease	1.1627
XAC_RS02840	XAC0545	3-deoxy-7-phosphoheptulonate synthase	-1.4464
XAC_RS22920	XAC0577	DNA cytosine methyltransferase	-1.5199
XAC_RS03065	XAC0590	DUF3817 domain-containing protein	1.9166
XAC_RS03165	XAC0607	hypothetical protein	-1.8517
XAC_RS03190	XAC0612	endoglucanase	1.5921
XAC_RS03240	XAC0623	DUF481 domain-containing protein	-2.5256
XAC_RS03265	XAC0629	ATPase AAA	-1.9683
XAC_RS03275	XAC0631	S9 family peptidase	-2.4877
XAC_RS03305	XAC0637	HslU--HslV peptidase proteolytic subunit	1.5845
XAC_RS03540	XAC0682	BON domain-containing protein	2.6005
XAC_RS03840	XAC0742	hypothetical protein	-6.4096
XAC_RS03895	XAC0753	hypothetical protein	1.3482
XAC_RS04230	XAC0822	hemin uptake protein HemP	6.3377
XAC_RS04235	XAC0823	TonB-dependent hemoglobin/transferrin/lactoferrin family receptor	7.5977
XAC_RS04240	XAC0824	hypothetical protein	6.7084
XAC_RS04270	XAC0830	TauD/TfdA family dioxygenase	-1.8161
XAC_RS04290	XAC0834	DNA-binding response regulator	2.6376
XAC_RS04295	XAC0835	sensor histidine kinase	2.4136
XAC_RS04550	XAC0888	gfo/Idh/MocA family oxidoreductase	1.4551
XAC_RS04625	XAC0900	peptide-methionine (S)-S-oxide reductase	2.3659
XAC_RS04650	XAC0906	alkyl hydroperoxide reductase subunit F	-1.2251
XAC_RS04700	XAC0916	alpha/beta hydrolase	-1.2489
XAC_RS04720	XAC0920	DUF3106 domain-containing protein	1.5524
XAC_RS04725	XAC0921	hypothetical protein	1.9038
XAC_RS04730	XAC0922	RNA polymerase sigma factor	2.3605
XAC_RS04760	XAC0928	peptidase S8	3.7961
XAC_RS04765	XAC0929	peptidase S8	2.5605
XAC_RS04770	XAC0930	peptidase S8	-2.8838
XAC_RS04815	XAC0940	hypothetical protein	-2.6645
XAC_RS04830	XAC0943	hypothetical protein	-1.2261
XAC_RS04835	XAC0944	peptide chain release factor 1	-1.4272
XAC_RS04840	XAC0945	glutamyl-tRNA reductase	-1.3942
XAC_RS05160	XAC1008	cell wall hydrolase	-2.1835
XAC_RS05290	XAC1034	peptidase	1.7038
XAC_RS05395	XAC1054	integrase	-1.846
XAC_RS05420	XAC1058	hypothetical protein	-3.4908

XAC_RS05425	XAC1059	antirestriction protein ArdA	-4.4472
XAC_RS05430	XAC1060	hypothetical protein	-5.6486
XAC_RS05485	XAC1072	phage-related DNA-directed RNA polymerase	-2.0307
XAC_RS05575	XAC1091	serine/threonine-protein phosphatase	-1.3386
XAC_RS05600	XAC1096	MBL fold metallo-hydrolase	-1.2535
XAC_RS05695	XAC1113	Starvation-inducible hypothetical protein	-3.1493
XAC_RS06010	XAC1178	SDR family NAD(P)-dependent oxidoreductase	2.3912
XAC_RS06045	XAC1184	EAL domain-containing protein	-1.5822
XAC_RS06105	XAC1196	LexA repressor 1	-1.9055
XAC_RS06110	XAC1197	DNA lesion error-prone repair protein ImuA	-1.7753
XAC_RS06130	XAC1201	HDOD domain-containing protein	-2.278
XAC_RS06220	XAC1219	phosphatase PAP2 family protein	-2.8578
XAC_RS06310	XAC1236	membrane protein	1.2641
XAC_RS06340	XAC1242	hypothetical protein	1.3941
XAC_RS06455	XAC1265	DNA-binding response regulator	-3.2289
XAC_RS06515	XAC1277	thioredoxin TrxC	1.2536
XAC_RS06520	XAC1278	DUF1428 domain-containing protein	1.8292
XAC_RS06640	XAC1300	RNA-binding S4 domain-containing protein	2.0583
XAC_RS06745	XAC1320	hypothetical protein	1.7927
XAC_RS06750	XAC1321	DegQ family serine endoprotease	2.2412
XAC_RS06830	XAC1337	cold-shock protein	1.3105
XAC_RS06875	XAC1345	GGDEF domain-containing protein	-1.213
XAC_RS06880	XAC1346	hypothetical protein	1.2996
XAC_RS06925	XAC1355	DksA/TraR family C4-type zinc finger protein	1.4847
XAC_RS07275	XAC1423	molecular chaperone	-2.9581
XAC_RS07340	XAC1435	TonB-dependent siderophore receptor	4.1681
XAC_RS07380	XAC1443	MarR family transcriptional regulator	2.9005
XAC_RS07385	XAC1444	multidrug RND transporter	1.4207
XAC_RS07405	XAC1448	glycosyl hydrolase	-2.3154
XAC_RS07455	XAC1458	ferredoxin--NADP reductase	1.4168
XAC_RS07465	XAC1460	Tartrate dehydratase alpha subunit	-2.0409
XAC_RS07490	XAC1465	cold-shock protein	3.4087
XAC_RS07495	XAC1466	glycine zipper 2TM domain-containing protein	-2.0122
XAC_RS07520	XAC1471	glycine zipper 2TM domain-containing protein	-6.2983
XAC_RS07550	XAC1476	NAD(P)H-dependent oxidoreductase	1.7845
XAC_RS07570	XAC1480	LysR family transcriptional regulator	1.3552

XAC_RS07580	XAC1481	SDR family NAD(P)-dependent oxidoreductase	1.4312
XAC_RS07585	XAC1482	MexE family multidrug efflux RND transporter periplasmic adaptor subunit	1.9013
XAC_RS07590	XAC1483	multidrug efflux RND transporter permease subunit	1.5906
XAC_RS07595	XAC1484	SDR family NAD(P)-dependent oxidoreductase	1.4209
XAC_RS25040	XAC1489	hypothetical protein	-1.5937
XAC_RS07625	XAC1492	hypothetical protein	1.4967
XAC_RS07680	XAC1509	DUF4209 domain-containing protein	1.2951
XAC_RS07725	XAC1517	ferric iron uptake transcriptional regulator	1.9532
XAC_RS07850	XAC1542	class II fumarate hydratase	1.1987
XAC_RS07910	XAC1554	transporter	1.4176
XAC_RS08035	XAC1580	carbonic anhydrase	-3.0024
XAC_RS08040	XAC1581	SulP family inorganic anion transporter	-1.4644
XAC_RS08320	XAC1635	urocanate hydratase	-1.249
XAC_RS08360	XAC1642	hypothetical protein	1.3115
XAC_RS08480	XAC1666	methyl-accepting chemotaxis protein	-2.0015
XAC_RS08905	XAC1748	LysR family transcriptional regulator	-1.259
XAC_RS09060	XAC1778	sensor kinase	1.7678
XAC_RS23395	XAC1779	hypothetical protein	1.5719
XAC_RS09135	XAC1795	sensor domain-containing phosphodiesterase	-4.033
XAC_RS09290	XAC1827	DNA-binding transcriptional regulator	-3.3049
XAC_RS09295	XAC1828	ATP phosphoribosyltransferase	-2.679
XAC_RS09385	XAC1846	DUF2388 domain-containing protein	-3.702
XAC_RS09390	XAC1847	DUF4105 domain-containing protein	-1.8156
XAC_RS09430	XAC1855	ferrous iron transporter B	1.2698
XAC_RS09595	XAC1886	2-succinyl-6-hydroxy-2%2C4-cyclohexadiene-1-carboxylate synthase	-1.5103
XAC_RS09630	XAC1893	methyl-accepting chemotaxis protein	-1.8642
XAC_RS09640	XAC1894	methyl-accepting chemotaxis protein	-3.876
XAC_RS10210	XAC2008	outer membrane lipoprotein carrier protein LolA	-1.6047
XAC_RS10240	XAC2013	3-hydroxyacyl-CoA dehydrogenase	1.0677
XAC_RS10300	XAC2025	hypothetical protein	3.3072
XAC_RS10305	XAC2026	DUF4142 domain-containing protein	3.1722
XAC_RS10310	XAC2027	hypothetical protein	3.2527
XAC_RS10315	XAC2028	glutathione-dependent formaldehyde dehydrogenase	2.6171
XAC_RS10325	XAC2030	exodeoxyribonuclease III	3.0442
XAC_RS10445	XAC2054	PAS domain S-box protein	-1.4784

XAC_RS10735	XAC2113	DUF3300 domain-containing protein	-3.016
XAC_RS10740	XAC2114	zinc transporter ZupT	1.7792
XAC_RS10850	XAC2135	hypothetical protein	2.2089
XAC_RS10900	XAC2144	autotransporter domain-containing protein	-3.3117
XAC_RS10930	XAC2151	outer membrane protein	6.0421
XAC_RS10935	XAC2152	phosphodiesterase	4.2433
XAC_RS10940	XAC2153	DUF1349 domain-containing protein	1.7681
XAC_RS10950	XAC2155	ferritin-like domain-containing protein	2.9833
XAC_RS11110	XAC2185	TonB-dependent siderophore receptor	1.2881
XAC_RS11255	XAC2215	AAA family ATPase	1.7298
XAC_RS11715	XAC2300	50S ribosomal protein L36	1.5441
XAC_RS11730	XAC2303	apolipoprotein acyltransferase	-1.4707
XAC_RS11775	XAC2312	membrane protein	-2.8585
XAC_RS11780	XAC2313	LacI family transcriptional regulator	-2.4666
XAC_RS11810	XAC2319	hypothetical protein	-1.2543
XAC_RS11885	XAC2334	thiol reductant ABC exporter subunit CydC	-1.6054
XAC_RS11890	XAC2335	thiol reductant ABC exporter subunit CydD	-1.5846
XAC_RS11895	XAC2336	cytochrome bd oxidase subunit I	-1.3264
XAC_RS11900	XAC2337	cytochrome d ubiquinol oxidase subunit II	-2.2447
XAC_RS11905	XAC2338	cytochrome bd-I oxidase subunit CydX	-2.2366
XAC_RS11980	XAC2353	hypothetical protein	-3.9221
XAC_RS12045	XAC2367	hypothetical protein	1.6402
XAC_RS12055	XAC2369	general stress protein	1.8412
XAC_RS12140	XAC2387	ribonuclease	-2.4211
XAC_RS12145	XAC2388	barstar family protein	-1.5721
XAC_RS12175	XAC2394	glutathione S-transferase	1.4801
XAC_RS12260	XAC2411	virulence factor	-4.288
XAC_RS12280	XAC2415	DUF3606 domain-containing protein	1.9365
XAC_RS12615	XAC2480	glutamine synthetase	1.4064
XAC_RS12675	XAC2492	hybrid sensor histidine kinase/response regulator	1.2139
XAC_RS12680	XAC2493	two-component system response regulator	1.4661
XAC_RS12695	XAC2497	TetR family transcriptional regulator	2.0944
XAC_RS12780	XAC2512	preprotein translocase subunit YajC	1.2503
XAC_RS13045	XAC2561	membrane protein	1.6811
XAC_RS13535	XAC2663	hypothetical protein	1.2717
XAC_RS22645	XAC2664	type IV pilin protein	1.9131
XAC_RS13540	XAC2665	pilus assembly protein	1.8421
XAC_RS22655	XAC2668	type IV pilus modification protein PilV	1.9397

XAC_RS13550	XAC2669	prepilin-type N-terminal cleavage/methylation domain-containing protein	1.7111
XAC_RS13670	XAC2694	NADH-quinone oxidoreductase subunit K	-1.4921
XAC_RS13745	XAC2709	group 1 truncated hemoglobin	1.3574
XAC_RS13755	XAC2711	sensor domain-containing phosphodiesterase	1.1026
XAC_RS13925	XAC2742	TonB-dependent receptor	2.5791
XAC_RS14075	XAC2772	SIMPL domain-containing protein	-1.6797
XAC_RS14130	XAC2783	thioredoxin	1.3633
XAC_RS14155	XAC2788	DUF378 domain-containing protein	3.8076
XAC_RS14180	XAC2793	hypothetical protein	2.212
XAC_RS14225	XAC2802	outer membrane channel protein	-2.0203
XAC_RS14260	XAC2808	DUF72 domain-containing protein	1.3456
XAC_RS14325	XAC2821	hypothetical protein	-2.2977
XAC_RS14435	XAC2843	multidrug efflux RND transporter permease subunit	1.5623
XAC_RS14440	XAC2844	MexE family multidrug efflux RND transporter periplasmic adaptor subunit	2.9365
XAC_RS14460	XAC2848	DUF3616 domain-containing protein	-1.2107
XAC_RS14560	XAC2868	diguanylate phosphodiesterase	1.1767
XAC_RS14565	XAC2869	protein-glutamate O-methyltransferase CheR	1.2954
XAC_RS14570	XAC2870	chemotaxis response regulator protein-glutamate methylesterase	1.125
XAC_RS14790	XAC2914	glycine zipper 2TM domain-containing protein	-6.2516
XAC_RS14795	XAC2915	OsmC family peroxiredoxin	1.8621
XAC_RS14845	XAC2924	type IV pili twitching motility protein PilT	1.0859
XAC_RS14880	XAC2931	DUF1439 domain-containing protein	-2.8284
XAC_RS14885	XAC2932	type 1 glutamine amidotransferase	2.7379
XAC_RS14895	XAC2934	SUF system Fe-S cluster assembly regulator	1.7064
XAC_RS14915	XAC2938	cysteine desulfurase	1.3877
XAC_RS14930	XAC2941	TonB-dependent siderophore receptor	5.3977
XAC_RS14935	XAC2942	PKHD-type hydroxylase	4.594
XAC_RS14940	XAC2943	sel1 repeat family protein	4.1439
XAC_RS14945	XAC2944	membrane protein	3.628
XAC_RS14950	XAC2945	DUF2271 domain-containing protein	4.2676
XAC_RS14955	XAC2946	DUF4198 domain-containing protein	4.7579
XAC_RS14960	XAC2947	FAD:protein FMN transferase	3.2421
XAC_RS14965	XAC2948	sulfite reductase	2.8884

XAC_RS15035	XAC2962	DUF3108 domain-containing protein	-1.7683
XAC_RS15155	XAC2986	pectate lyase	1.3415
XAC_RS15185	XAC2992	serine protease	2.8034
XAC_RS15200	XAC2995	tryptophan 7-halogenase	1.3356
XAC_RS15205	XAC2996	cupin-like domain-containing protein	1.1591
XAC_RS15215	XAC2998	TonB-dependent receptor	1.2183
XAC_RS15315	XAC3017	glycerol-3-phosphate dehydrogenase subunit C	1.7605
XAC_RS15420	XAC3037	alpha/beta hydrolase	1.7816
XAC_RS15450	XAC3043	hemolysin D	2.1633
XAC_RS15475	XAC3048	Hsp33 family molecular chaperone HslO	1.4457
XAC_RS15485	XAC3050	TonB-dependent receptor	2.885
XAC_RS15580	XAC3070	glucokinase	-2.9748
XAC_RS15585	XAC3071	TonB-dependent receptor	-3.2034
XAC_RS15665	XAC3087	RebB protein	1.183
XAC_RS15705	XAC3096	chemotaxis protein CheW	1.319
XAC_RS15825	XAC3121	TonB-dependent receptor	1.4211
XAC_RS15845	XAC3125	hypothetical protein	-4.1655
XAC_RS15855	XAC3128	hypothetical protein	1.6893
XAC_RS15945	XAC3146	tol-pal system-associated acyl-CoA thioesterase	-1.3064
XAC_RS15995	XAC3155	DUF3011 domain-containing protein	-4.926
XAC_RS16105	XAC3177	siderophore biosynthesis protein PvsA	3.3037
XAC_RS16110	XAC3178	IucA/IucC family siderophore biosynthesis protein	3.3431
XAC_RS16115	XAC3179	MFS transporter	2.166
XAC_RS16120	XAC3180	iron transporter	3.4558
XAC_RS16125	XAC3181	siderophore biosynthesis PLP-dependent protein	3.4684
XAC_RS16215	XAC3199	glyceraldehyde 3-phosphate reductase	1.7023
XAC_RS16240	XAC3204	DUF3014 domain-containing protein	2.5571
XAC_RS16255	XAC3208	hypothetical protein	1.347
XAC_RS16440	XAC3240	pilin	1.6424
XAC_RS16995	XAC3354	OmpW family protein	-1.4564
XAC_RS17005	XAC3356	endonuclease	-1.3657
XAC_RS17070	XAC3369	hypothetical protein	1.4459
XAC_RS17125	XAC3379	MoxR family ATPase	-1.3867
XAC_RS17410	XAC3439	hypothetical protein	-4.2272
XAC_RS17435	XAC3444	TonB-dependent receptor	-7.1241
XAC_RS17440	XAC3445	AraC family transcriptional regulator	-4.2626
XAC_RS17445	XAC3446	biopolymer transporter Tol	-1.782
XAC_RS17770	XAC3515	cellulose synthase operon protein C	-1.2598
XAC_RS17780	XAC3517	divalent ion tolerance protein CutA	-1.4328

XAC_RS17855	XAC3532	UTRA domain-containing protein	-1.3316
XAC_RS17860	XAC3533	hypothetical protein	1.2661
XAC_RS17865	XAC3534	type II secretion system protein GspD	1.3744
XAC_RS18000	XAC3560	TonB-dependent receptor	1.1916
XAC_RS18310	XAC3620	TonB-dependent siderophore receptor	3.285
XAC_RS18365	XAC3632	lactoylglutathione lyase	1.3689
XAC_RS18525	XAC3664	membrane protein	-7.2159
XAC_RS18620	XAC3680	hypothetical protein	1.414
XAC_RS18650	XAC3686	DUF3016 domain-containing protein	-2.2437
XAC_RS18675	XAC3691	peptide-methionine (R)-S-oxide reductase	1.6081
XAC_RS18760	XAC3709	flavodoxin family protein	-1.242
XAC_RS18775	XAC3712	metallopeptidase	-1.6434
XAC_RS18780	XAC3714	DUF2236 domain-containing protein	-1.503
XAC_RS18825	XAC3725	ferritin-like domain-containing protein	3.916
XAC_RS18830	XAC3726	Mn-containing catalase	1.9992
XAC_RS18840	XAC3728	hypothetical protein	2.1913
XAC_RS18855	XAC3731	response regulator	2.5754
XAC_RS18890	XAC3739	hypothetical protein	2.3569
XAC_RS18910	XAC3742	UDP-galactopyranose mutase	1.4257
XAC_RS18925	XAC3745	DUF3606 domain-containing protein	1.5866
XAC_RS18930	XAC3746	DUF4142 domain-containing protein	2.0698
XAC_RS18935	XAC3747	glutathione-dependent formaldehyde dehydrogenase	2.5937
XAC_RS19170	XAC3797	O-antigen ligase family protein	-1.6358
XAC_RS19415	XAC3846	NADPH-dependent 7-cyano-7-deazaguanine reductase QueF	-1.6454
XAC_RS19435	XAC3850	AcrB/AcrD/AcrF family protein	-1.2887
XAC_RS19455	XAC3854	haloacid dehalogenase	-1.27
XAC_RS19465	XAC3856	hypothetical protein	-5.0063
XAC_RS19515	XAC3866	hypothetical protein	3.5134
XAC_RS19525	XAC3868	PQQ-dependent sugar dehydrogenase	1.7211
XAC_RS19530	XAC3869	beta-glucosidase	-1.3432
XAC_RS19780	XAC3921	glycosyltransferase	1.9423
XAC_RS19785	XAC3922	non-ribosomal peptide synthetase	2.0072
XAC_RS19990	XAC3966	hypothetical protein	1.3429
XAC_RS20000	XAC3969	DUF3182 domain-containing protein	2.7426
XAC_RS20015	XAC3971	hypothetical protein	3.5434
XAC_RS20020	XAC3972	DUF4142 domain-containing protein	2.292
XAC_RS20050	XAC3979	histidine biosynthesis protein HisIE	-1.4637
XAC_RS20080	XAC3986	HAD family hydrolase	-1.1987
XAC_RS20220	XAC4014	thymidylate kinase	-1.2161
XAC_RS20225	XAC4015	tRNA-Thr	-2.9711
XAC_RS20255	XAC4020	DUF1501 domain-containing protein	-1.6807

XAC_RS20260	XAC4021	DUF1800 domain-containing protein	-2.867
XAC_RS20270	XAC4023	DNA-binding response regulator	#NAME?
XAC_RS20275	XAC4024	hypothetical protein	-2.1763
XAC_RS20285	XAC4026	hypothetical protein	-3.4438
XAC_RS20305	XAC4031	ATP-dependent DNA helicase DinG	-1.1121
XAC_RS20330	XAC4036	iron-uptake factor	1.9025
XAC_RS20340	XAC4038	LysR family transcriptional regulator	-1.2757
XAC_RS20545	XAC4078	magnesium transporter	-2.3392
XAC_RS20695	XAC4107	hypothetical protein	-1.313
XAC_RS20720	XAC4112	type VI secretion system protein TssA	2.0747
XAC_RS20725	XAC4113	filamentous hemagglutinin N-terminal domain-containing protein	2.573
XAC_RS20730	XAC4114	ShlB/FhaC/HecB family hemolysin secretion/activation protein	3.1209
XAC_RS20830	XAC4135	hypothetical protein	-1.3804
XAC_RS21005	XAC4169	GlsB/YeaQ/YmgE family stress response membrane protein	-1.1623
XAC_RS21075	XAC4182	cytochrome c biogenesis protein	5.0765
XAC_RS21140	XAC4192	membrane protein	-2.489
XAC_RS21255	XAC4214	GMP synthase	-2.0457
XAC_RS21280	XAC4219	hypothetical protein	-2.1635
XAC_RS21775	XAC4318	hypothetical protein	1.2755
XAC_RS21840	XAC4330	hypothetical protein	-1.8781
XAC_RS21970	XAC4357	DUF2628 domain-containing protein	-1.2749

Table S4. List of genes differentially expressed in the *phoP* mutant supplemented with 0.05 M NaCl compared to the *phoP* mutant (Log2 fold change ≥ 0.9).

Gene ID	Gene name	Gene function	Log2 fold change
XAC_RS00130	XAC0025	hypothetical protein	0.94717
XAC_RS00335	XAC0065	phage tail protein	-1.0916
XAC_RS00340	XAC0066	phage tail protein	-1.0314
XAC_RS00345	XAC0067	phage tail protein	-1.0219
XAC_RS00570	XAC0110	proline/glycine betaine transporter ProP	2.0759
XAC_RS00775	XAC0147	hypothetical protein	-1.1274
XAC_RS00885	XAC0170	hypothetical protein	0.92155
XAC_RS01070	XAC0204	type I glutamate--ammonia ligase	1.6295
XAC_RS01075	XAC0205	P-II family nitrogen regulator	1.3303
XAC_RS01215	XAC0233	3-oxoacyl-ACP synthase III	0.99223
XAC_RS01745	XAC0334	FMN reductase	-1.1326
XAC_RS01765	XAC0338	porin	-1.5433
XAC_RS01820	XAC0345	dihydroxy-acid dehydratase	0.90077
XAC_RS02045	XAC0388	biotin synthase	1.1089
XAC_RS02350	XAC0449	di-tripeptide transporter	1.3817
XAC_RS02450	XAC0470	Phosphoribosylaminoimidazole-succinocarboxamide synthase	1.1111
XAC_RS02535	XAC0487	50S ribosomal protein L13	1.1104
XAC_RS02540	XAC0488	30S ribosomal protein S9	1.1464
XAC_RS02565	XAC0493	bacterioferritin	-1.0446
XAC_RS02765	XAC0531		1.0628
XAC_RS02770	XAC0532	acetyl-CoA carboxylase biotin carboxyl carrier protein	0.96916
XAC_RS02825	XAC0542	molecular chaperone GroEL	0.99469
XAC_RS02920	XAC0560	malonate decarboxylase subunit alpha	1.1023
XAC_RS02970	XAC0570	anti-sigma factor antagonist	-1.1699
XAC_RS02985	XAC0573	hypothetical protein	1.2543
XAC_RS03000	XAC0576	pyruvate dehydrogenase subunit E1	1.4376
XAC_RS03125	XAC0600	D-serine/D-alanine/glycine transporter	1.4816
XAC_RS03275	XAC0631	S9 family peptidase	1.0297
XAC_RS03405	XAC0656	rod shape-determining protein	1.3186
XAC_RS03410	XAC0657	rod shape-determining protein MreC	1.1611
XAC_RS03705	XAC0716	TonB-dependent receptor	1.2693
XAC_RS03885	XAC0751	N utilization substance protein B	1.126
XAC_RS03895	XAC0753	hypothetical protein	-2.0239
XAC_RS04155	XAC0806	phosphoenolpyruvate carboxylase	0.91685

XAC_RS04280	XAC0832	ABC transporter ATP-binding protein	-1.0108
XAC_RS04780	XAC0932	asparaginase	0.93691
XAC_RS04815	XAC0940	hypothetical protein	-1.3561
XAC_RS04865	XAC0950	ribose-phosphate pyrophosphokinase	1.3015
XAC_RS04870	XAC0951	50S ribosomal protein L25	1.3477
XAC_RS04875	XAC0952	peptidyl-tRNA hydrolase	1.6233
XAC_RS04905	XAC0958	tRNA	1.8097
XAC_RS04910	XAC0959	preprotein translocase subunit SecE	1.5802
XAC_RS04925	XAC0962	50S ribosomal protein L1	1.0065
XAC_RS04930	XAC0963	50S ribosomal protein L10	1.0983
XAC_RS04950	XAC0967	30S ribosomal protein S12	1.2167
XAC_RS04955	XAC0968	30S ribosomal protein S7	1.2108
XAC_RS04975	XAC0972	50S ribosomal protein L3	1.218
XAC_RS04980	XAC0973	50S ribosomal protein L4	1.2809
XAC_RS04985	XAC0974	50S ribosomal protein L23	1.5999
XAC_RS04995	XAC0976	30S ribosomal protein S19	1.252
XAC_RS05015	XAC0980	50S ribosomal protein L29	1.7149
XAC_RS05020	XAC0981	30S ribosomal protein S17	0.91748
XAC_RS05045	XAC0986	30S ribosomal protein S8	0.95555
XAC_RS05050	XAC0987	50S ribosomal protein L6	1.0772
XAC_RS05055	XAC0988	50S ribosomal protein L18	0.93581
XAC_RS05085	XAC0994	30S ribosomal protein S11	1.102
XAC_RS05090	XAC0995	30S ribosomal protein S4	1.0912
XAC_RS05135	XAC1003	DUF2127 domain-containing protein	1.2926
XAC_RS05140	XAC1004	translational GTPase TypA	1.036
XAC_RS05155	XAC1007	glutathione S-transferase	0.96193
XAC_RS05255	XAC1028	histidine phosphatase family protein	1.0298
XAC_RS05420	XAC1058	hypothetical protein	1.5157
XAC_RS05425	XAC1059	antirestriction protein ArdA	1.7784
XAC_RS05430	XAC1060	hypothetical protein	3.2617
XAC_RS05490	XAC1073	tRNA	1.0935
XAC_RS05505	XAC1077	trigger factor	0.90073
XAC_RS05535	XAC1083	tRNA	1.8035
XAC_RS05545	XAC1085	peptidylprolyl isomerase	1.1819
XAC_RS05580	XAC1092	tRNA	1.6125
XAC_RS05730	XAC1120	septum formation inhibitor Maf	0.97915
XAC_RS05755	XAC1126	[acyl-carrier-protein] S-malonyltransferase	1.5145
XAC_RS05760	XAC1127	3-oxoacyl-ACP reductase FabG	1.8127
XAC_RS06030	XAC1181	alpha-ketoglutarate-dependent dioxygenase AlkB	-1.4751

XAC_RS06125	XAC1200	S9 family peptidase	1.8214
XAC_RS06215	XAC1218	phosphoethanolamine transferase	-1.1376
XAC_RS06270	XAC1228	sensor histidine kinase	0.91274
XAC_RS06340	XAC1242	hypothetical protein	-1.0501
XAC_RS06370	XAC1248	50S ribosomal protein L21	0.99249
XAC_RS06375	XAC1249	50S ribosomal protein L27	1.0216
XAC_RS06380	XAC1250	GTPase ObgE	0.92447
XAC_RS06385	XAC1251	30S ribosomal protein S20	1.23
XAC_RS06440	XAC1262	peptidase M61	1.3648
XAC_RS06465	XAC1267	molecular chaperone HtpG	1.3583
XAC_RS06470	XAC1268	hypothetical protein	1.1863
XAC_RS06545	XAC1282	PAS domain-containing sensor histidine kinase	1.0117
XAC_RS06640	XAC1300	RNA-binding S4 domain-containing protein	-1.1879
XAC_RS06730	XAC1317	cation transporter	-1.0206
XAC_RS06790	XAC1329	23S rRNA (uracil(1939)-C(5))-methyltransferase RlmD	1.0129
XAC_RS06870	XAC1344	cell shape determination protein CcmA	-1.2722
XAC_RS06925	XAC1355	DksA/TraR family C4-type zinc finger protein	-1.203
XAC_RS06945	XAC1357	heat-shock protein Hsp70	1.3943
XAC_RS06950	XAC1358	peptidylprolyl isomerase	1.3161
XAC_RS06960	XAC1360	phosphonoacetate hydrolase	1.2825
XAC_RS07105	XAC1389	ABC transporter ATP-binding protein	1.1261
XAC_RS07125	XAC1393	DUF885 domain-containing protein	0.9629
XAC_RS07140	XAC1396	hypothetical protein	1.1053
XAC_RS07180	XAC1404	hypothetical protein	0.92515
XAC_RS07265	XAC1421	elongation factor Ts	1.5017
XAC_RS07270	XAC1422	30S ribosomal protein S2	1.1548
XAC_RS07405	XAC1448	glycosyl hydrolase	0.90947
XAC_RS07445	XAC1456	M3 family peptidase	1.4318
XAC_RS07465	XAC1460	Tartrate dehydratase alpha subunit	0.92164
XAC_RS07580	XAC1481	SDR family NAD(P)-dependent oxidoreductase	-2.1884
XAC_RS07585	XAC1482	MexE family multidrug efflux RND transporter periplasmic adaptor subunit	-1.7474
XAC_RS07590	XAC1483	multidrug efflux RND transporter permease subunit	-1.0729
XAC_RS07595	XAC1484	SDR family NAD(P)-dependent oxidoreductase	-1.0399

XAC_RS07600	XAC1485	RND transporter	-1.0719
XAC_RS07625	XAC1492	hypothetical protein	-1.1358
XAC_RS23265	XAC1501	hypothetical protein	1.2968
XAC_RS23270	XAC1503	hypothetical protein	1.0825
XAC_RS08010	XAC1576	phosphate ABC transporter permease subunit PstC	-1.6394
XAC_RS08015	XAC1577	phosphate ABC transporter substrate-binding protein PstS	-1.0302
XAC_RS08250	XAC1620	30S ribosomal protein S6	1.1535
XAC_RS08255	XAC1621	30S ribosomal protein S18	1.3683
XAC_RS08260	XAC1622	50S ribosomal protein L9	1.3815
XAC_RS08285	XAC1627	DNA ligase (NAD(+)) LigA	0.98722
XAC_RS08370	XAC1644	polyhydroxyalkanoic acid synthase	1.1127
XAC_RS08390	XAC1648	phosphoserine aminotransferase	0.92535
XAC_RS08395	XAC1649	prephenate dehydratase	0.97656
XAC_RS08570	XAC1682	DNA-directed RNA polymerase sigma-70 factor	-1.4931
XAC_RS08590	XAC1685	hypothetical protein	-1.9851
XAC_RS08645	XAC1696	class I SAM-dependent methyltransferase	-2.5199
XAC_RS08665	XAC1700	glycosyltransferase family 1 protein	-2.8633
XAC_RS08920	XAC1751	tRNA	2.3117
XAC_RS08995	XAC1766	2-dehydro-3-deoxy-6-phosphogalactonate aldolase	-1.576
XAC_RS09200	XAC1808	aldehyde dehydrogenase family protein	0.97857
XAC_RS09290	XAC1827	DNA-binding transcriptional regulator	2.3671
XAC_RS09360	XAC1841	amino acid permease	1.2196
XAC_RS09365	XAC1842	amino acid permease	1.3279
XAC_RS09375	XAC1844	phosphoglycerate dehydrogenase	1.0019
XAC_RS09380	XAC1845	FAD-binding oxidoreductase	1.0332
XAC_RS09605	XAC1888	chemotaxis response regulator protein-glutamate methylesterase	-1.0155
XAC_RS09630	XAC1893	methyl-accepting chemotaxis protein	-1.2054
XAC_RS09640	XAC1894	methyl-accepting chemotaxis protein	-1.0432
XAC_RS09645	XAC1895	methyl-accepting chemotaxis protein	-1.2021
XAC_RS09655	XAC1897	chemotaxis protein	-1.292
XAC_RS09690	XAC1902	methyl-accepting chemotaxis protein	-1.1066
XAC_RS10495	XAC2064	efflux RND transporter periplasmic	1.2557

		adaptor subunit	
XAC_RS10500	XAC2065	AcrB/AcrD/AcrF family protein	1.1489
XAC_RS10565	XAC2078	succinate dehydrogenase iron-sulfur subunit	0.91769
XAC_RS10580	XAC2081	lipoprotein-releasing system transmembrane subunit LolC	0.93222
XAC_RS10775	XAC2121	O-methyltransferase	-1.1593
XAC_RS10930	XAC2151	outer membrane protein	-1.2235
XAC_RS10935	XAC2152	phosphodiesterase	-1.0003
XAC_RS11210	XAC2206	hypothetical protein	-3.1514
XAC_RS11475	XAC2257	integrating conjugative element protein	-2.3822
XAC_RS11565	XAC2273	TIGR03752 family integrating conjugative element protein	-1.1558
XAC_RS11625	XAC2284	integrating conjugative element protein	-1.9499
XAC_RS11655	XAC2288	IMP dehydrogenase	1.0961
XAC_RS11705	XAC2298	30S ribosomal protein S1	1.1231
XAC_RS11710	XAC2299	cytidylate kinase	1.0963
XAC_RS11715	XAC2300	50S ribosomal protein L36	0.93419
XAC_RS11740	XAC2305	TraB family protein	0.97162
XAC_RS11775	XAC2312	membrane protein	1.1076
XAC_RS11890	XAC2335	thiol reductant ABC exporter subunit CydD	0.94595
XAC_RS11920	XAC2341	glutaryl-7-ACA acylase	1.9572
XAC_RS11940	XAC2345	argininosuccinate lyase	1.1361
XAC_RS11945	XAC2346	N-acetyl-gamma-glutamyl-phosphate reductase	1.0991
XAC_RS11955	XAC2348	acetylglutamate kinase	1.1498
XAC_RS11960	XAC2349	acetylornithine deacetylase	1.1216
XAC_RS11965	XAC2350	GNAT family N-acetyltransferase	1.3745
XAC_RS11970	XAC2351	argininosuccinate synthase	1.3603
XAC_RS11975	XAC2352	acetylornithine carbamoyltransferase	1.5373
XAC_RS11990	XAC2355	hypothetical protein	1.0705
XAC_RS11995	XAC2356	MFS transporter	1.1184
XAC_RS12130	XAC2385	inositol monophosphatase	1.0313
XAC_RS12200	XAC2399	protease HtpX	-1.7685
XAC_RS12515	XAC2461	adenosine tRNA methylthiotransferase	0.905
XAC_RS12665	XAC2490	hypothetical protein	1.361
XAC_RS12910	XAC2535	TonB-dependent receptor	-1.4591
XAC_RS12975	XAC2548	FAD-dependent oxidoreductase	-1.9218
XAC_RS13005	XAC2553	A/G-specific adenine glycosylase	0.98485

XAC_RS13180	XAC2588	integration host factor subunit alpha	1.1648
XAC_RS13185	XAC2589	phenylalanine--tRNA ligase subunit beta	1.2676
XAC_RS13190	XAC2590	phenylalanine--tRNA ligase subunit alpha	1.1814
XAC_RS13195	XAC2591	50S ribosomal protein L20	1.0748
XAC_RS13200	XAC2592	50S ribosomal protein L35	1.2172
XAC_RS13250	XAC2602	alpha-glucosidase	1.0626
XAC_RS13535	XAC2663	hypothetical protein	-1.5048
XAC_RS22645	XAC2664	type IV pilin protein	-1.0029
XAC_RS13540	XAC2665	pilus assembly protein	-1.0383
XAC_RS13640	XAC2688	transcription elongation factor NusA	0.9977
XAC_RS13650	XAC2690	tRNA	1.1946
XAC_RS13660	XAC2692	NADH-quinone oxidoreductase subunit M	0.95709
XAC_RS13665	XAC2693	NADH-quinone oxidoreductase subunit L	0.92259
XAC_RS13670	XAC2694	NADH-quinone oxidoreductase subunit K	1.1863
XAC_RS13675	XAC2695	NADH-quinone oxidoreductase subunit J	1.16
XAC_RS13680	XAC2696	NADH-quinone oxidoreductase subunit I	1.1374
XAC_RS13685	XAC2697	NADH-quinone oxidoreductase subunit H	1.0669
XAC_RS13690	XAC2698	NADH dehydrogenase (quinone) subunit G	0.94474
XAC_RS13695	XAC2699	NADH-quinone oxidoreductase subunit F	1.0813
XAC_RS13700	XAC2700	NADH-quinone oxidoreductase subunit NuoE	1.1502
XAC_RS13705	XAC2701	NADH-quinone oxidoreductase subunit D	1.2937
XAC_RS13710	XAC2702	NADH-quinone oxidoreductase subunit C	1.2108
XAC_RS13860	XAC2729	lytic transglycosylase	1.3195
XAC_RS13940	XAC2745	peptidase	1.4257
XAC_RS14085	XAC2774	energy transducer TonB	-1.1982
XAC_RS14095	XAC2776	DUF839 domain-containing protein	-1.242
XAC_RS14225	XAC2802	outer membrane channel protein	-1.0798
XAC_RS14355	XAC2827	30S ribosomal protein THX	0.93355
XAC_RS14435	XAC2843	multidrug efflux RND transporter permease subunit	0.94673

XAC_RS14545	XAC2865	chemotaxis protein CheA	-1.0398
XAC_RS14550	XAC2866	methyl-accepting chemotaxis protein	-1.2295
XAC_RS14555	XAC2867	chemotaxis protein CheW	-1.0418
XAC_RS14775	XAC2911	diaminopimelate decarboxylase	0.9721
XAC_RS14850	XAC2925	YggS family pyridoxal phosphate-dependent enzyme	0.95898
XAC_RS14885	XAC2932	type 1 glutamine amidotransferase	1.0962
XAC_RS14955	XAC2946	DUF4198 domain-containing protein	-1.0234
XAC_RS14980	XAC2951	DNA transport competence protein	0.96283
XAC_RS15135	XAC2982	cytochrome d ubiquinol oxidase subunit II	1.3632
XAC_RS15140	XAC2983	cytochrome ubiquinol oxidase subunit I	1.0949
XAC_RS15160	XAC2987	amino acid amidase	-1.7512
XAC_RS15300	XAC3014	glycerol-3-phosphate dehydrogenase subunit C	-1.4353
XAC_RS15340	XAC3021	hypothetical protein	-1.0217
XAC_RS15350	XAC3023	DUF1906 domain-containing protein	-1.1348
XAC_RS15445	XAC3042	peptide chain release factor 3	1.401
XAC_RS15580	XAC3070	glucokinase	1.436
XAC_RS15655	XAC3085	hypothetical protein	-1.0526
XAC_RS15665	XAC3087	RebB protein	-1.2223
XAC_RS15825	XAC3121	TonB-dependent receptor	-1.227
XAC_RS15970	XAC3150	crossover junction endodeoxyribonuclease RuvC	0.99396
XAC_RS15975	XAC3151	YebC/PmpR family DNA-binding transcriptional regulator	0.9662
XAC_RS15990	XAC3154	aspartate--tRNA ligase	1.221
XAC_RS16000	XAC3156	zinc ribbon domain-containing protein	1.3819
XAC_RS16190	XAC3194	TonB-dependent vitamin B12 receptor	1.105
XAC_RS16240	XAC3204	DUF3014 domain-containing protein	1.1562
XAC_RS16775	XAC3311	TonB-dependent receptor	1.94
XAC_RS16780	XAC3312	beta-galactosidase	1.9391
XAC_RS16790	XAC3314	DUF239 domain-containing protein	1.1492
XAC_RS16810	XAC3318	aminopeptidase	1.1487
XAC_RS17170	XAC3389	50S ribosomal protein L31 type B	1.2606
XAC_RS17210	XAC3397	ribonuclease PH	0.99285
XAC_RS17220	XAC3399	deoxyribonucleotide triphosphate	1.1639

		pyrophosphatase	
XAC_RS17225	XAC3400	YggW family oxidoreductase	1.0817
XAC_RS17415	XAC3440	K+-insensitive pyrophosphate-energized proton pump	-1.591
XAC_RS17440	XAC3445	AraC family transcriptional regulator	-1.6334
XAC_RS17585	XAC3474	MFS transporter	-1.4837
XAC_RS17920	XAC3545	protease	1.2206
XAC_RS18145	XAC3589	membrane protein	1.2871
XAC_RS18150	XAC3590	FAD-binding oxidoreductase	1.1728
XAC_RS18325	XAC3623	beta-hydroxydecanoyl-ACP dehydratase	1.8489
XAC_RS18330	XAC3625	beta-ketoacyl-[acyl-carrier-protein] synthase I	1.5798
XAC_RS18620	XAC3680	hypothetical protein	-1.218
XAC_RS18670	XAC3690	hypothetical protein	-1.477
XAC_RS18795	XAC3717	APC family permease	0.96628
XAC_RS18855	XAC3731	response regulator	-1.5043
XAC_RS24345	XAC3785	DUF4102 domain-containing protein	1.2263
XAC_RS19120	XAC3788	RNA polymerase sigma factor RpoD	0.93114
XAC_RS19235	XAC3810	hypothetical protein	-1.0433
XAC_RS19395	XAC3842	type I-C CRISPR-associated endonuclease Cas1	-2.1223
XAC_RS19545	XAC3872	30S ribosomal protein S21	1.1993
XAC_RS19630	XAC3890	bifunctional proline dehydrogenase/L-glutamate gamma-semialdehyde dehydrogenase PutA	-1.0036
XAC_RS19665	XAC3898	membrane protein	0.98715
XAC_RS19980	XAC3964	hypothetical protein	-1.6981
XAC_RS19995	XAC3967	alpha/beta hydrolase	1.0215
XAC_RS20295	XAC4028	ankyrin repeat domain-containing protein	-1.9807
XAC_RS20370	XAC4044	DUF885 domain-containing protein	1.071
XAC_RS20685	XAC4105	AMP-ligase	1.3025
XAC_RS20690	XAC4106	S9 family peptidase	1.5348
XAC_RS20720	XAC4112	type VI secretion system protein TssA	-1.1697
XAC_RS20725	XAC4113	filamentous hemagglutinin N-terminal domain-containing protein	-1.196
XAC_RS20730	XAC4114	ShlB/FhaC/HecB family hemolysin secretion/activation protein	-1.3582

XAC_RS20745	XAC4117	serine/threonine-protein phosphatase	-4.0897
XAC_RS20750	XAC4118	type VI secretion system-associated protein TagF	-3.1075
XAC_RS20795	XAC4127	serine/threonine protein kinase	-1.0621
XAC_RS20830	XAC4135	hypothetical protein	0.97503
XAC_RS20935	XAC4156	4-oxalomesaconate tautomerase	-1.6365
XAC_RS21075	XAC4182	cytochrome c biogenesis protein	-1.3859
XAC_RS21305	XAC4223	recombination-associated protein RdgC	0.99129
XAC_RS21385	XAC4239	MFS transporter	-1.6001
XAC_RS21415	XAC4246	DUF2147 domain-containing protein	-2.8047
XAC_RS21695	XAC4302	GTP cyclohydrolase I FolE	1.2534
XAC_RS24660	XAC4356	hypothetical protein	1.1817
XAC_RS21980	XAC4359	CdaR family transcriptional regulator	-1.2572
XAC_RS22015	XAC4366	rhomboid family intramembrane serine protease	-1.6116
XAC_RS22055	XAC4374	50S ribosomal protein L34	1.4344
XAC_RS22255	XACb0003	hypothetical protein	0.92169

Table S5. List of genes differentially expressed in the *phoP* mutant supplemented with 0.05 M sorbitol compared to the *phoP* mutant (Log2 fold change ≥ 1).

Gene ID	Gene name	Gene function	Log_2 fold change
XAC_RS04730	XAC0922	RNA polymerase sigma factor	-1.248
XAC_RS06330	XAC1240	hypothetical protein	-1.3783
XAC_RS07405	XAC1448	glycosyl hydrolase	1.1134
XAC_RS07445	XAC1456	M3 family peptidase	1.1584
XAC_RS10500	XAC2065	AcrB/AcrD/AcrF family protein	1.1894
XAC_RS11775	XAC2312	membrane protein	1.563
XAC_RS11920	XAC2341	glutaryl-7-ACA acylase	1.44
XAC_RS12200	XAC2399	protease HtpX	-1.0676
XAC_RS16775	XAC3311	TonB-dependent receptor	2.3107
XAC_RS16780	XAC3312	beta-galactosidase	2.0435
XAC_RS16790	XAC3314	DUF239 domain-containing protein	1.3582
